# Darkness Attenuates the Early Transcriptional Response to Phosphate Deficiency in Soybean Roots

**DOI:** 10.3390/ijms27177963

**Published:** 2026-09-07

**Authors:** Anamta Shaikh, Izabel Thurber, Nikko R. M. Sacramento, Jennifer Bravo, Lynne Viall, Reemaben Maniyar, Maria Muhammad Ali, Jennifer Nguyen, Kristine Tran, Geronimo Parra, Kayla Magdaleno, Trinidad Cruz, Kamilah Baltrons, Rafael Cazares, Brandon DeLeon, Nathan Flores, Monika Sommerhalter, Claudia Uhde-Stone

**Affiliations:** 1 Department of Biological Sciences, California State University, East Bay, Hayward, CA 94542, USAmali60@horizon.csueastbay.edu (M.M.A.);; 2 Department of Chemistry and Biochemistry, California State University, East Bay, Hayward, CA 94542, USAbdeleon2@horizon.csueastbay.edu (B.D.); monika.sommerhalter@csueastbay.edu (M.S.); 3 Post-Baccalaureate Health Professions Academic Program (PHAP), California State University, East Bay, Hayward, CA 94542, USA; rcazares4@horizon.csueastbay.edu

**Keywords:** carbon availability, *Glycine max*, Nanopore sequencing, phosphate-starvation response, phosphate deficiency, RNA-seq, sucrose

## Abstract

Phosphate (P_i_) deficiency induces responses that enhance P_i_ uptake and utilization. Light may influence these responses through photosynthetic carbon supply and signaling. We examined how light affects the early root transcriptional response to P_i_ deficiency in hydroponically grown soybean. Plants were exposed to phosphate-sufficient (+P) or phosphate-deficient (−P) conditions for 30 h under a light/dark cycle or continuous darkness. Root transcriptomes were analyzed using Oxford Nanopore cDNA sequencing. Principal component analysis (PCA) showed that transcriptomes separated mainly by light, with weaker separation by P_i_ status. Under light, P_i_ deficiency induced a broad response involving P_i_ transport, signaling, transcriptional regulation, lipid remodeling, and metabolism. In darkness, relatively few genes were upregulated under P_i_ deficiency. This limited response was not caused by a loss of transcriptional responsiveness, because thousands of genes were upregulated by darkness itself. Comparison of fold changes revealed that darkness attenuated, rather than reversed, the −P response. Interaction-ranked gene set enrichment analysis (GSEA) showed that the weaker response in darkness extended across signaling, metabolic, and transport pathways. Light-signaling genes responded strongly to darkness but showed little phosphate-dependent regulation. Root sucrose levels were lower in darkness, consistent with reduced carbon availability as a possible contributor to the attenuated P_i_-deficiency response.

## 1. Introduction

Phosphorus (P) is an essential macronutrient for all living organisms. As a component of nucleic acids, phospholipids, and ATP, P supports the structural and energetic foundations of life and plays key roles in energy transfer, membrane stability, and signaling in plant cells [1]. Most P is taken up as inorganic phosphate (P_i_), but in many soils P_i_ is tightly bound to minerals or organic matter, limiting its availability for uptake [1]. To sustain crop productivity, large amounts of P fertilizer are applied, most of it mined from non-renewable geological deposits formed over millions of years. However, intensive mining and fertilizer use have led to declining global reserves and to environmental pollution through runoff and soil erosion [2,3,4].

Improving plant P acquisition and utilization is therefore critical for sustainable agriculture. Soybean (*Glycine max*), a major source of plant-based protein and oil [5], is particularly sensitive to P limitation because biological nitrogen fixation in root nodules requires high P availability to support the large ATP demand associated with nitrogenase activity and nodule metabolism [6]. In soybean, progress toward improving P efficiency has come from quantitative trait loci (QTL) analyses identifying loci associated with acid phosphatase activity and root-associated P acquisition traits [7] and molecular and transcriptomic studies of P_i_ transporters, PHR signaling components, and metabolic adaptation to P deficiency [8,9,10]. These studies have revealed diverse responses to P deficiency, including changes in root architecture and exudation [11], altered leaf P-allocation, and improved P-use efficiency [9].

Under P deficiency, root system architecture changes, typically showing shorter primary roots and more lateral roots and root hairs, which increase the root surface area exposed to soil P_i_ [11,12]. Roots also secrete organic acids such as citrate and malate that mobilize P_i_, and release phosphatases and nucleases that free P_i_ from organic compounds [1,11,13]. At the cellular level, high-affinity P_i_ transporters are upregulated to improve uptake efficiency [14,15,16], while membrane lipid remodeling conserves P by shifting from phospholipids to P_i_-free membrane lipids [17]. Central carbon metabolism also becomes more P_i_-efficient by favoring PP_i_-dependent reactions that bypass ATP [18,19].

Although P_i_ deficiency affects the whole plant, transcriptional and metabolic responses are initiated by changes in cellular P_i_ availability and coordinated through P_i_-responsive signaling networks. SPX-domain proteins and PHR transcription factors form a core regulatory module [20]. Under P_i_-sufficient conditions, SPX proteins bind inositol pyrophosphates and repress PHR activity; under P_i_ deficiency, this repression is relieved, allowing PHRs to activate P_i_-responsive genes [21]. Soybean homologs such as GmPHR1, GmSPX1, and GmSPX3 modulate P_i_ uptake and homeostasis [22], while PHO1 family members export P_i_ into the xylem and contribute to whole-plant P_i_ distribution [23].

Light influences plant nutrient responses through both photosynthesis-dependent and signaling pathways. In addition to driving carbon assimilation and the supply of photosynthate to roots, light is perceived through photoreceptors that regulate transcriptional networks controlling growth, metabolism, and nutrient acquisition [24]. In Arabidopsis, the photomorphogenesis regulator HY5 can act as a shoot-to-root mobile signal and has been implicated in coordinating light and carbon status with root nutrient responses, including responses to P_i_ availability [25]. Red-light signaling through phytochrome B also regulates P_i_ acquisition through PIF4/PIF5 and HY5, which modulate P_i_-responsive genes [26]. In addition, FHY3, FAR1, and HY5 regulate *PHR1* expression and phosphate-starvation responses [27]. Light may therefore affect the root phosphate-starvation response directly, through transcriptional and signaling pathways that interact with nutrient-responsive networks, or more indirectly, by altering the supply of photosynthetically derived carbon.

One important component of this shoot-to-root communication is sucrose. Sucrose, the main product of photosynthesis, functions both as a carbon source and as a signal integrating plant carbon status with nutrient responses [28,29,30]. Under P_i_ deficiency, increased sucrose transport from leaves to roots stimulates the expression of P_i_-acquisition genes, including high-affinity phosphate transporters and acid phosphatases [31,32]. In soybean, sucrose can trigger rapid transcriptomic changes within minutes, activating hormone, calcium, and ROS signaling before canonical P_i_-starvation genes respond [33]. Other systemic signals, including auxin, ethylene, strigolactones, and microRNAs such as miR399 and miR827, further integrate carbon status with P_i_ allocation and root development [34,35,36,37]. Several of these pathways are themselves influenced by carbon and sucrose signaling [29,30,31].

P_i_ status can also be sensed locally in roots. Root tips and epidermal tissues respond to P_i_ deficiency through mechanisms involving LPR1/LPR2-dependent Fe redox chemistry, auxin transport, reactive oxygen species, and hormone signaling [38,39]. Thus, the root phosphate-starvation response reflects the integration of local P_i_ sensing with systemic signals derived from the shoot.

However, how continuous darkness affects the early phosphate-starvation transcriptional response in soybean roots has not been characterized. In this study, we used Oxford Nanopore cDNA sequencing to analyze soybean root responses to 30 h of P_i_ withdrawal under a standard light/dark cycle or continuous darkness. We predicted that P_i_ deficiency under the standard light/dark cycle would induce a broad early phosphate-starvation response and asked whether, and to what extent, this response would be reduced during continuous darkness.

## 2. Results

### 2.1. Nanopore Sequencing Provides Sufficient Depth and Transcriptome Coverage for Downstream Analyses

**Figure 1:**
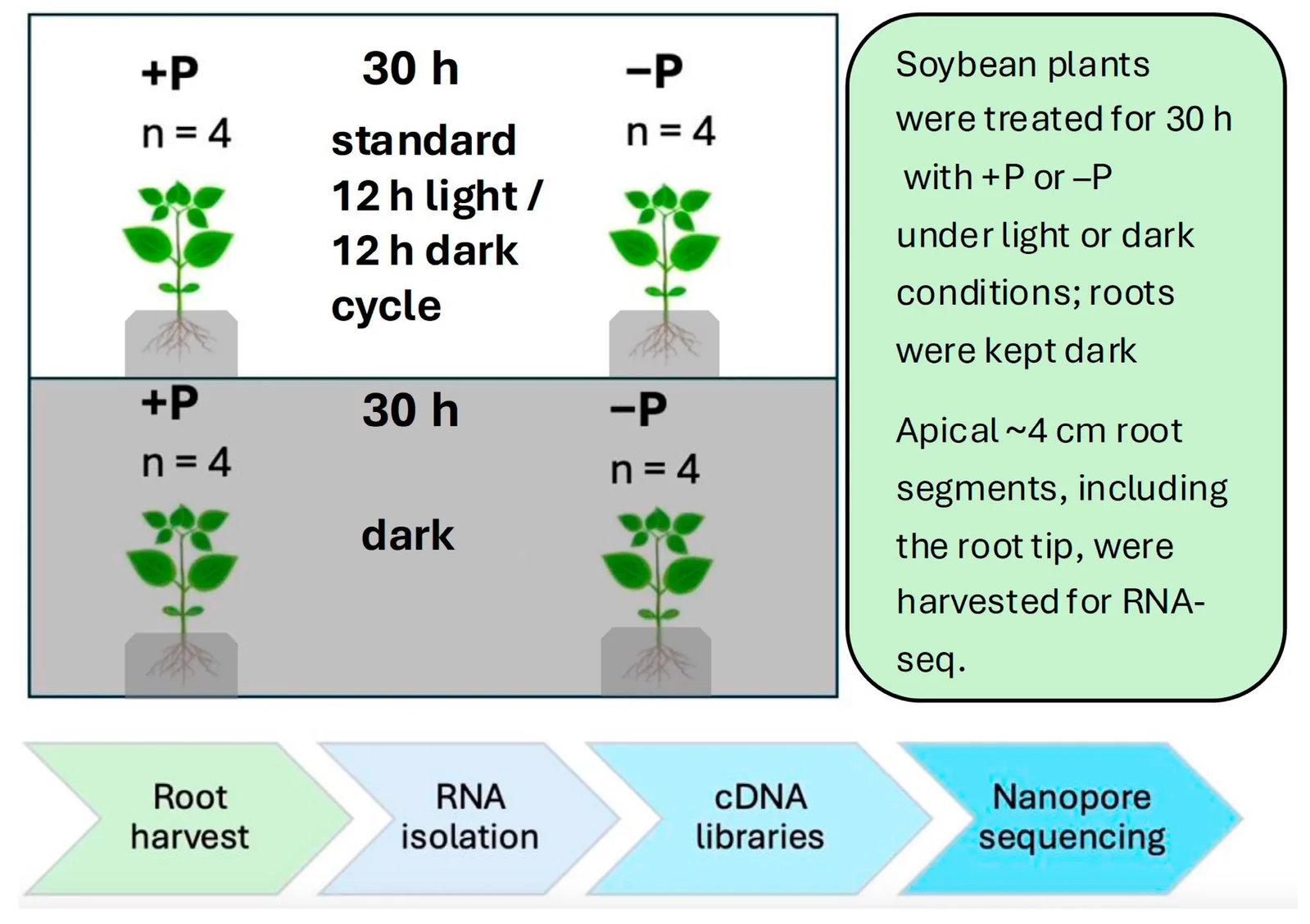
Experimental design. Soybean plants were grown hydroponically for two weeks and then exposed for 30 h to phosphate-sufficient (+P) or phosphate-deficient (−P) conditions under either a standard light/dark cycle or continuous darkness, while roots were maintained in darkness in all treatments. Each treatment included four biological replicates, with one plant per treatment sampled in each of four independent hydroponic experimental blocks. ~4 cm apical root segments, including the root tip, were harvested for RNA isolation, cDNA library preparation, and Oxford Nanopore sequencing.

Soybean plants were grown hydroponically for two weeks and then exposed for 30 h to phosphate-sufficient (+P) or phosphate-deficient (−P) conditions under either a standard light/dark cycle or continuous darkness, while roots were maintained in darkness in all treatments (Figure 1). Each treatment included four biological replicates.

Oxford Nanopore cDNA sequencing generated a mean of 4.8–6.9 × 10^6^ passed reads per sample across treatment groups, with mean quality scores of Q10–11 and N50 values of approximately 800–1000 bp (Table 1; see Table S1 for additional sequencing and mapping metrics). Across all samples, sequencing yielded approximately 90 million passed reads, with a mean of 5.6 million reads per sample and mapping efficiencies exceeding 90%. After filtering low-abundance genes by requiring at least 10 raw reads in at least three replicates within any treatment group, 25,348 genes were retained for downstream analyses. These represented approximately 54% of the 47,068 protein-coding genes annotated in the *Glycine max* v4.0 genome. Taken together, the sequencing metrics indicate that the sequencing depth and transcriptome coverage were sufficient to support downstream comparisons across treatments.

### 2.2. Light Condition Is the Main Source of Transcriptomic Variation, While the P_i_ Response Is Strongly Reduced in Darkness

**Figure 2:**
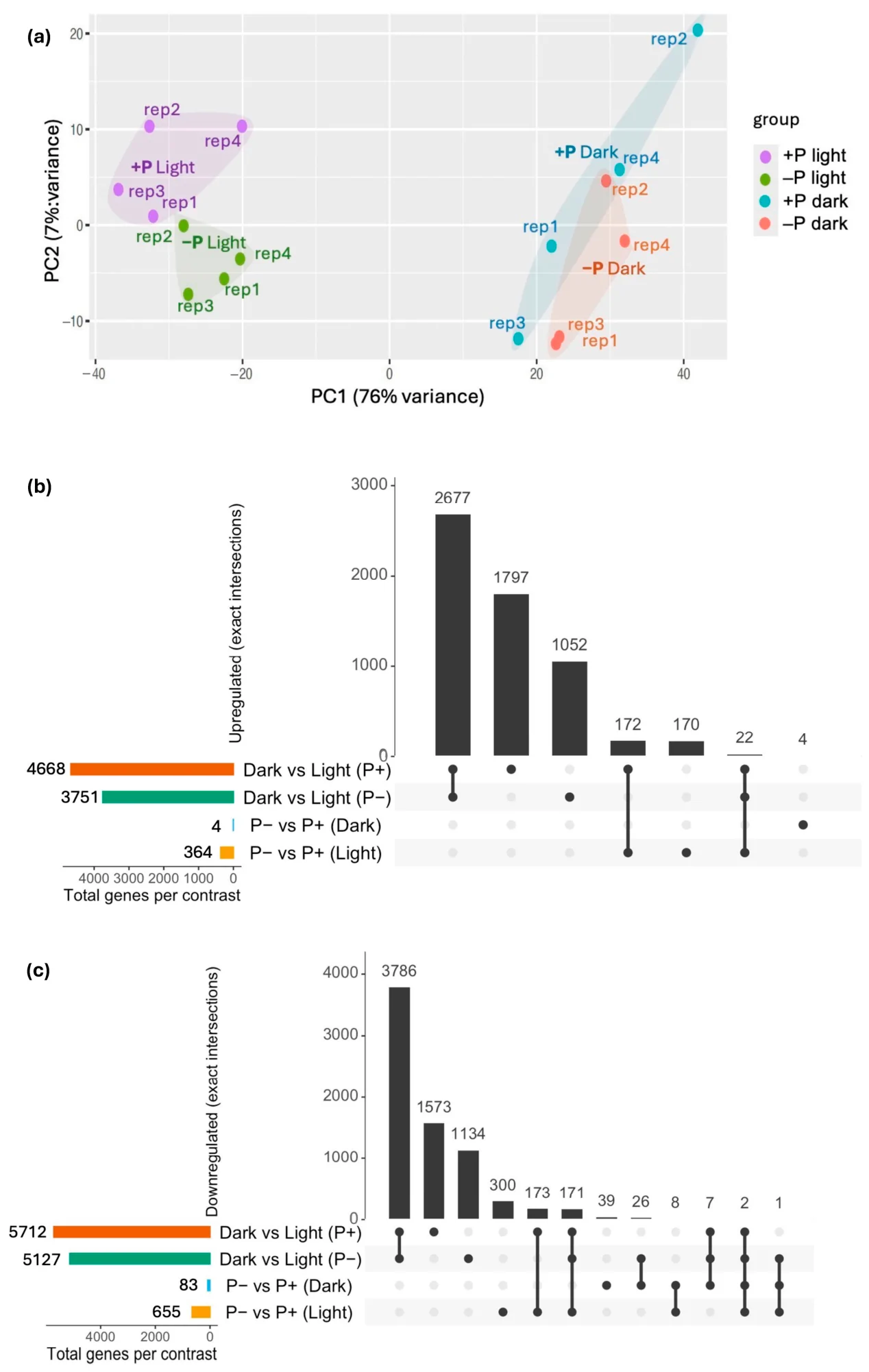
Darkness causes widespread changes in gene expression and reduces the response to −P. (**a**) PCA of normalized gene-expression data. PC1 explained 76% of the variance and separated light-grown from dark-grown samples, while PC2 explained 7% of the variance and showed a smaller separation associated with P_i_ status. Under light conditions, +P and −P samples formed distinct clusters, whereas the P_i_ treatments were less clearly separated in darkness. (**b**,**c**) UpSet plots show intersections among genes significantly upregulated (**b**) or downregulated (**c**) in the four pairwise comparisons, using an adjusted *p*-value < 0.05 and |log_2_FC| > 1. Horizontal bars indicate the total number of differentially expressed genes in each comparison, and vertical bars indicate the size of exact intersections among comparisons. Colors indicate the four treatment comparisons shown in the labels. Thousands of genes were differentially expressed between dark- and light-grown samples under both +P and −P conditions, showing a strong transcriptional response to darkness. In contrast, substantially fewer genes responded to −P in darkness than under light.

Principal component analysis (PCA; Figure 2a) showed that samples separated primarily by light condition. PC1 explained 76% of the variance and separated light-grown from dark-grown samples. A smaller separation by P_i_ status was visible along PC2, which explained 7% of the variance, particularly under light conditions. Under light, +P and −P samples separated more clearly, whereas they overlapped in darkness, consistent with a markedly reduced transcriptional response to P_i_ deficiency under dark conditions.

UpSet plots summarized the numbers and overlaps of up- and downregulated genes across comparisons (Figure 2b,c). Darkness caused extensive transcriptional reprogramming under both P_i_ treatments. In particular, 2677 genes were upregulated in darkness under both +P and −P conditions, while 3786 genes were downregulated under both conditions.

In roots from light-grown plants, 364 genes were upregulated and 655 were downregulated in response to −P. In darkness, only four genes were upregulated and 83 were downregulated using the standard significance thresholds (padj < 0.05, |log_2_FC| > 1). Together, these results show substantially fewer genes meeting the differential-expression thresholds in response to P_i_ deficiency in darkness than under light. The large numbers of upregulated genes in response to darkness itself indicate that this limited −P response is not caused by a general inability to change gene expression in the dark. We therefore used direct fold-change comparisons and light × phosphate interaction analyses to test whether the P_i_ response differed between light conditions.

**Figure 3:**
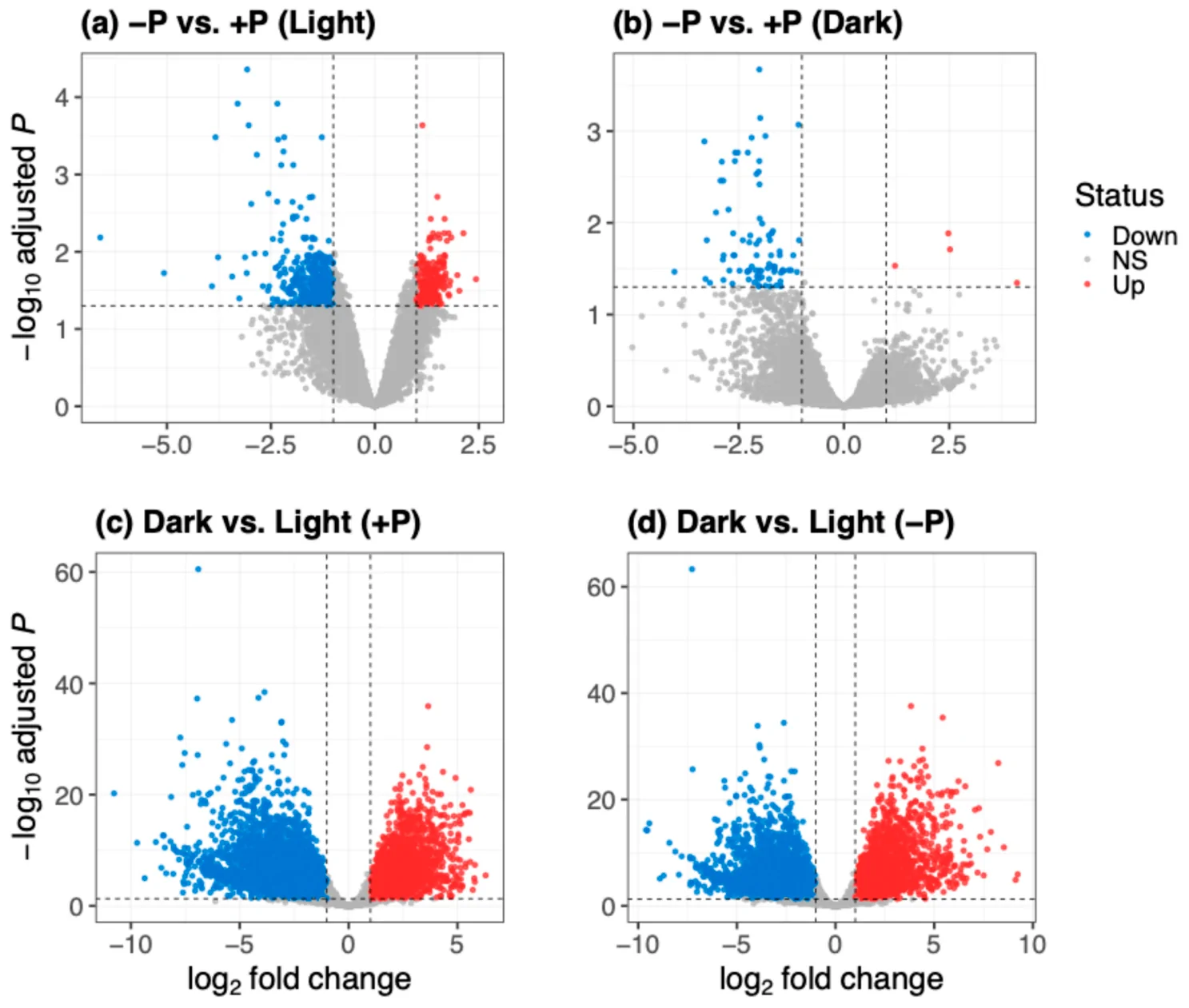
Volcano plots showing transcriptional responses to −P under light and dark conditions. (**a**–**d**) Volcano plots show the magnitude and statistical significance of expression changes for each comparison, using thresholds of adjusted *p*-values < 0.05 and |log_2_FC| > 1. Dashed lines indicate these significance thresholds. Red and blue points represent significantly up- and downregulated genes, respectively, whereas gray points are not significant. Panel (**a**) shows a strong transcriptional response to −P under light, whereas panel (**b**) shows only a limited −P response in darkness. Panels (**c**,**d**) show extensive differences in gene expression between light and darkness under both +P and −P conditions.

We next used volcano plots to examine the magnitude and statistical support of individual expression changes (Figure 3). Under light, −P treatment induced extensive transcriptional changes, with hundreds of genes significantly up- or downregulated. In darkness, however, only a small number of genes passed the significance thresholds, illustrating the markedly reduced transcriptomic response to −P in the dark.

### 2.3. Phosphate-Starvation Response Genes Are Induced Under Light but Show Limited Induction in Darkness

**Figure 4:**
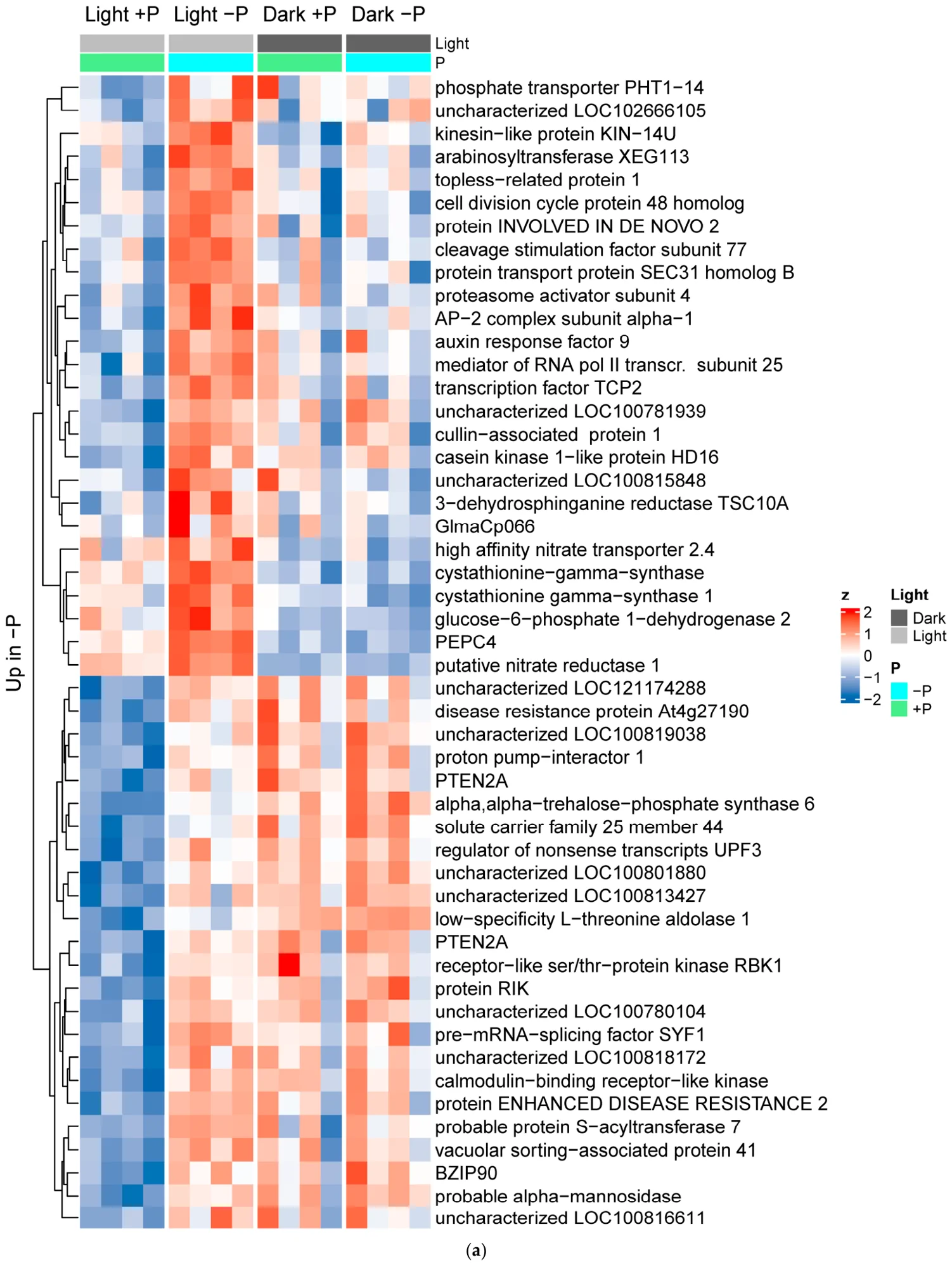

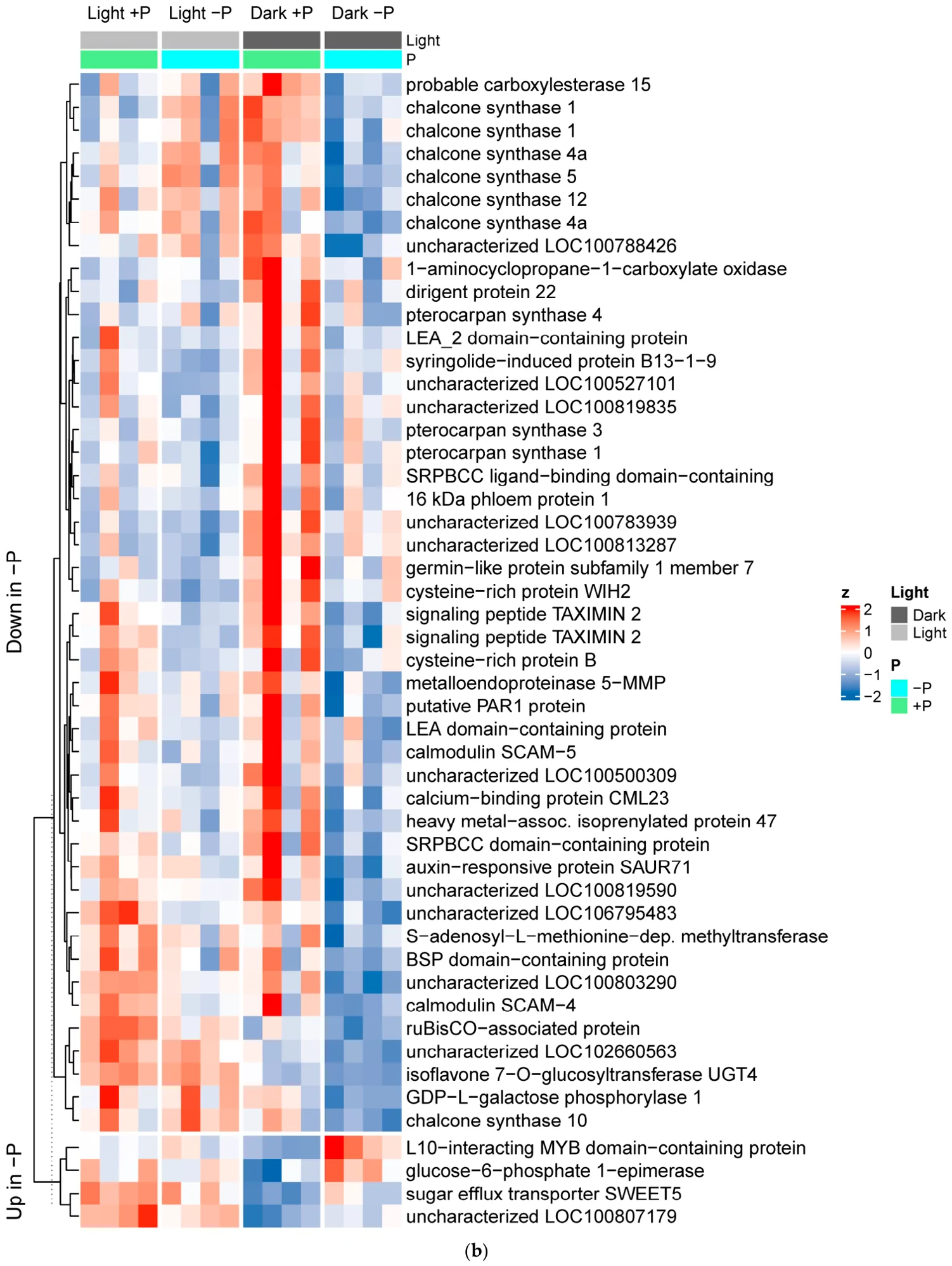
Differential expression in response to P_i_ deficiency under light and dark conditions. (**a**) Heatmap of the 50 most significantly upregulated genes in −P versus +P under light conditions selected from genes with adjusted *p* < 0.05 and log2 FC > 1 and ranked by adjusted *p*-value. Several well-characterized −P-responsive genes were strongly induced, including a high-affinity P_i_ transporter and genes associated with signaling and lipid remodeling. (**b**) Heatmap of the most differentially expressed genes in −P versus +P under dark conditions. Only four genes were significantly upregulated by −P, whereas numerous genes were downregulated. Rows represent individual genes, and columns represent biological replicates. Expression values are shown as z-score-normalized counts, with blue and red indicating lower and higher relative expression within each gene, respectively. Column annotations indicate phosphate status (+P or −P) and light condition.

To examine how transcriptional responses to P_i_ deficiency differed between light and dark conditions, we generated heatmaps of the genes most strongly affected by −P in each treatment (Figure 4 and Figure S1). Under light, roots showed clear induction of phosphate-starvation markers. Among the 50 most significantly upregulated genes was *PHT1*, encoding a high-affinity P_i_ transporter. Additional markers were present within the broader set of 364 genes upregulated by −P under light, including *PHO1* (phosphate exporter involved in xylem loading) and *SAP* (secreted acid phosphatase).

Only four genes were significantly upregulated by −P in darkness, including an L10-interacting MYB domain-containing transcription factor previously associated with stress responses [40]. In contrast, numerous stress-associated and metabolic genes, most notably many chalcone synthases, were downregulated by −P in darkness (Figure 4b). Together, these results show that P_i_-deficiency-induced transcriptional changes were strongly reduced in roots from dark-grown plants.

To determine whether the limited −P induction in darkness could be explained by low transcript abundance, we examined normalized counts for selected well-established phosphate-starvation response genes (Table 2). Markers such as *PHT1* and *PHO1* showed strong induction by −P under light but little or no change in darkness. Importantly, all genes examined in Table 2 exceeded the filtering threshold of at least 10 raw reads in at least three replicates, including in dark-grown samples. For example, *PHO1* had mean normalized counts of 24 in +P dark roots and 32 in −P dark roots, while *PHT1* had mean normalized counts of 944 and 807, respectively. Thus, the lack of a strong −P response in darkness was not due simply to failure to detect these transcripts.

### 2.4. The Reduced P_i_ Response in Darkness Reflects Attenuation Rather than Reversal

To determine whether the reduced −P response in darkness primarily reflects attenuation or a reversal of expression patterns, we compared the −P-associated log_2_ fold changes in genes significantly induced under light across both light and dark conditions (Figure S2). These genes had a median log_2_ fold change of 1.25 under light but only 0.14 in darkness, and only 0.55% retained a log_2_ fold change greater than 1 under dark conditions. Most genes showed little response in darkness rather than a strong response in the opposite direction.

Consistent with this overall pattern, a formal light × phosphate interaction analysis identified only 11 genes with significant interaction effects (Table S2). Several chalcone synthase genes were induced by −P under light but repressed in darkness, indicating that directional reversal occurred in a limited subset of genes. These analyses show that darkness mainly attenuated the −P response, whereas clear reversal was limited to a small number of genes.

Because broad attenuation could leave many genes with weaker responses that fail the standard cutoff, we next asked whether the minimal P_i_ response observed in darkness depended on the statistical thresholds. We therefore reanalyzed the dark dataset using relaxed criteria (|log_2_FC| > 1, adjusted *p* < 0.1). Using this approach, 13 genes were identified as upregulated, including the original four (Table 3; Figure S3). Genes upregulated under relaxed stringency included *PHR11*, two L10-interacting MYB domain–containing transcription factors, and several genes related to sugar metabolism. Of these 13 candidates, only one gene, *KIN-14U*, was also significantly induced under −P in light. Further relaxation of the criteria (|log_2_FC| > 0.5, and adjusted *p* < 0.1) identified only one additional P_i_-responsive gene, indicating that the broad attenuation observed in darkness was not simply a consequence of stringent statistical filtering.

We also tested whether the limited −P response in darkness depended on the differential-expression method. DESeq2 and other established count-based approaches have been used successfully for gene-level differential-expression analysis of Oxford Nanopore RNA-seq data [41]. As an alternative method developed specifically for long-read RNA-seq data, we used LRDE [42]. LRDE identified more differentially expressed genes than DESeq2 in both light and darkness, but the strong attenuation of the −P response in darkness remained. LRDE identified 982 genes upregulated by −P under light compared with 46 in darkness. The two methods showed substantial agreement for genes upregulated under light: 337 of the 364 genes upregulated by −P under light in the DESeq2 analysis were also identified by LRDE. In darkness, two of the four genes meeting the standard DESeq2 criteria and seven of the 13 genes identified using the relaxed criterion were also significantly upregulated by −P in the LRDE analysis (Table 3, Table 4 and Table S3). The genes supported by both methods are indicated in Table 3 by an asterisk.

### 2.5. Pathway-Level Responses to P_i_ Deficiency Are Broad Under Light but Attenuated in Darkness

**Figure 5:**
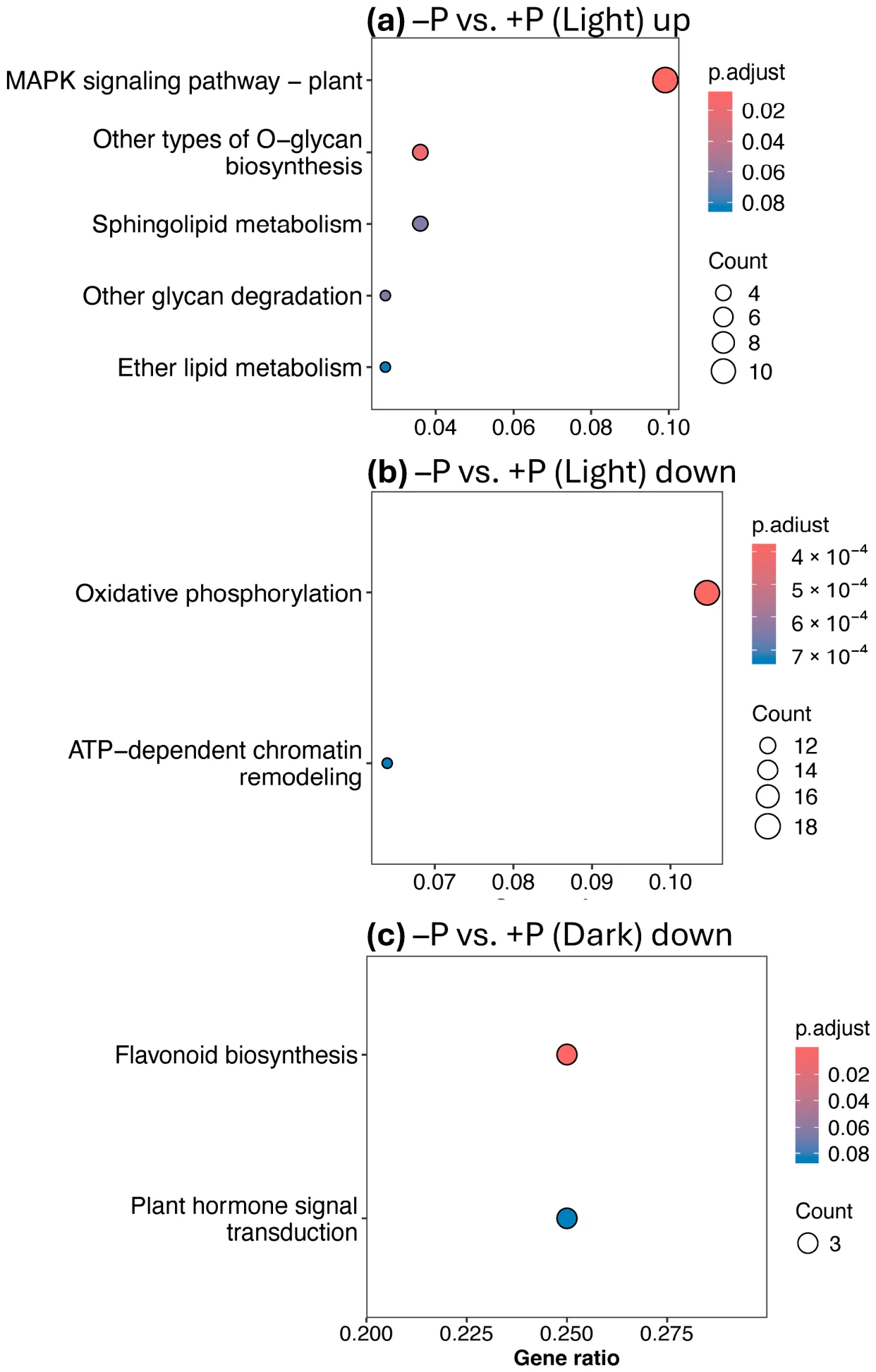
KEGG enrichment of genes differentially expressed under −P in light and darkness. Pathways are shown using an adjusted *p*-value cutoff of < 0.1 and requiring at least three DEGs to map to the pathway. (**a**) Pathways enriched among genes upregulated under −P in light (364 genes). Under light, MAPK signaling and lipid metabolism-related pathways were enriched among −P-responsive genes, whereas no enriched pathways meeting these criteria were identified among genes upregulated in darkness. (**b**,**c**) Pathways enriched among genes downregulated under −P in light (655 genes) or darkness (83 genes).

To characterize the biological processes associated with the transcriptional response to P_i_ deficiency, we first performed KEGG over-representation analysis of genes meeting the differential-expression thresholds. Under light, genes upregulated by P_i_ deficiency were enriched in MAPK signaling and lipid-related processes (Figure 5a). KEGG enrichment of the LRDE light-upregulated genes again identified MAPK signaling, while plant hormone signal transduction also reached significance in the LRDE analysis but was just above the adjusted-p threshold in the DESeq2 analysis.

Genes downregulated under P_i_ deficiency in light were enriched in oxidative phosphorylation and ATP-dependent chromatin remodeling (Figure 5b). In darkness, pathway enrichment was much more limited, consistent with the strongly attenuated transcriptional response. No KEGG pathways met the reporting criteria among the four genes upregulated by P_i_ deficiency in darkness in the DESeq2 analysis. Similarly, no KEGG pathways were identified among the 46 genes upregulated by P_i_ deficiency in darkness in the LRDE analysis. The downregulated set showed enrichment in flavonoid biosynthesis and plant hormone signaling (Figure 5c).

We also asked whether transcription factors were overrepresented among the Pi-responsive genes. Among the 364 genes induced by P_i_ deficiency under light, 30 were annotated as transcription factors representing multiple families, with ARF and bHLH the most frequently represented. However, transcription factors as a group were not significantly overrepresented relative to the expressed-gene background. Transcription factors were likewise not significantly overrepresented among the four genes induced in darkness.

Point size represents the number of genes assigned to each pathway, and color represents the adjusted *p*-value.

**Figure 6:**
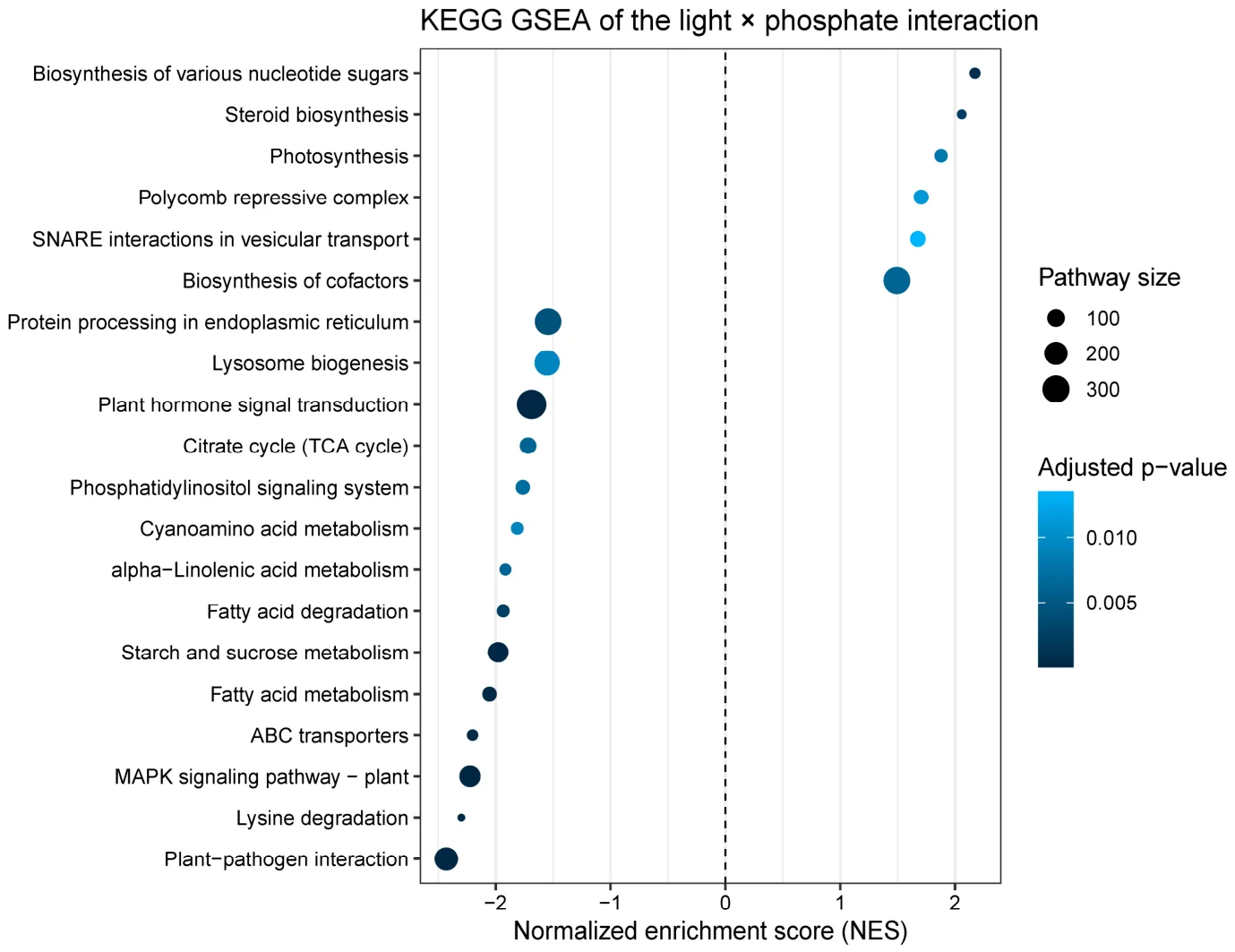
KEGG gene set enrichment analysis comparing the −P response under light and darkness. Genes were ranked according to the direction and strength of the difference in their −P response between darkness and light (signed Wald statistic for the interaction term). Negative normalized enrichment scores indicate pathways with a weaker −P response in darkness than in light, whereas positive scores indicate a stronger response in darkness. For pathways induced by −P under light, negative scores are consistent with attenuation of that response in darkness. Point size represents pathway size, and color indicates the Benjamini–Hochberg-adjusted *p* value.

Over-representation analysis includes only genes that pass the differential-expression cutoff. Because threshold-based analyses may miss coordinated changes among genes with smaller effects, we next used interaction-ranked GSEA to evaluate the full ranked transcriptome. Although only 11 genes had significant light × phosphate interaction effects, GSEA detected coordinated changes involving many genes with smaller effects. This pathway-level analysis therefore provided broader evidence for attenuation of the P_i_ response in darkness than was apparent from individually significant interaction genes alone. Most significant pathways showed a weaker P_i_ response in darkness than in light, consistent with attenuation of the response. These included plant MAPK signaling, hormone signaling, starch and sucrose metabolism, ABC transporters, plant–pathogen interaction, and several metabolic and vacuolar/endomembrane pathways. The KEGG “lysosome biogenesis” category consisted mainly of plant vacuolar and endomembrane-trafficking genes. A smaller number of pathways, including photosynthesis and nucleotide-sugar biosynthesis, showed positive enrichment scores (Figure 6). For these pathways, P_i_ deficiency caused a smaller decrease, or a greater increase, in gene expression in darkness than under light.

KEGG enrichment analysis of the light–dark comparison additionally showed that photosynthesis-, energy-, and metabolism-related pathways were major components of the broader response to darkness (Supplementary Figure S4).

### 2.6. Light-Signaling Genes Respond to Darkness but Show Limited Phosphate-Dependent Regulation

GO Biological Process enrichment was also examined across the major treatment comparisons (Figure S5). As with the KEGG analysis, broad changes were observed in metabolic, stress-response, photosynthesis-related, and other processes, but no discrete light-signaling category was significantly enriched.

We therefore examined genes annotated to light-signaling-related GO Biological Process terms directly. Fifty-one such genes passed the low-abundance expression filter and were included in the differential-expression analysis (Table S4). To confirm that this GO-based set captured major light-signaling components relevant to phosphate responses, we additionally examined established light-signaling genes using NCBI Gene annotations and BLAST searches. Several major components identified in this way, including *HY5*-related genes and genes associated with phytochrome, cryptochrome, and far-red signaling, were represented among the 51 genes. These annotations and gene identities are provided in Table S4.

Of the 51 light-signaling genes, 20 were differentially expressed between dark and light under +P and 24 under −P, with 17 genes shared between the two phosphate conditions. These included phytochrome- and cryptochrome-related genes, far-red signaling components, and several B-box transcriptional regulators. Ten genes met the dark-versus-light differential-expression criteria under only one phosphate condition: three under +P and seven under −P (Table 5). All 10 were upregulated in darkness. However, none of these genes, and none of the 51 light-signaling genes overall, showed a significant light × phosphate interaction after multiple-testing correction.

Because *FAR1* and *FHY3* are of particular interest in phosphate-starvation signaling [27] but were not included among the 51 genes identified from light-signaling-related GO terms, we examined their soybean homologs separately. BLAST searches identified LOC100814153 as the closest soybean homolog of *FAR1* and LOC100817034 as the closest homolog of *FHY3*. LOC100817034 was significantly upregulated in darkness under −P but not +P, whereas LOC100814153 showed a similar increase under −P but did not meet the adjusted-p threshold. Neither gene showed a significant light × phosphate interaction.

LOC100808627, encoding a cryptochrome-2-like protein, was significantly induced by −P under light (log_2_FC = 1.18, adjusted *p* = 0.017), whereas none of the 51 light-signaling genes was significantly responsive to −P in darkness. Thus, darkness broadly affected light-signaling gene expression, whereas phosphate-dependent regulation was limited, with the cryptochrome-2-like gene representing a notable −P-responsive component under light.

### 2.7. Protein–Protein Interaction Analysis Identifies Functional Clusters Among −P-Responsive Genes Under Light

We used STRING as an exploratory analysis to examine functional associations among the 364 genes induced by −P. The resulting network was partitioned using the Markov Cluster Algorithm (MCL). Each displayed cluster showed significantly more interactions than expected by chance, with cluster-specific PPI enrichment *p*-values ranging from <1 × 10^−4^ to <1 × 10^−16^.

**Figure 7:**
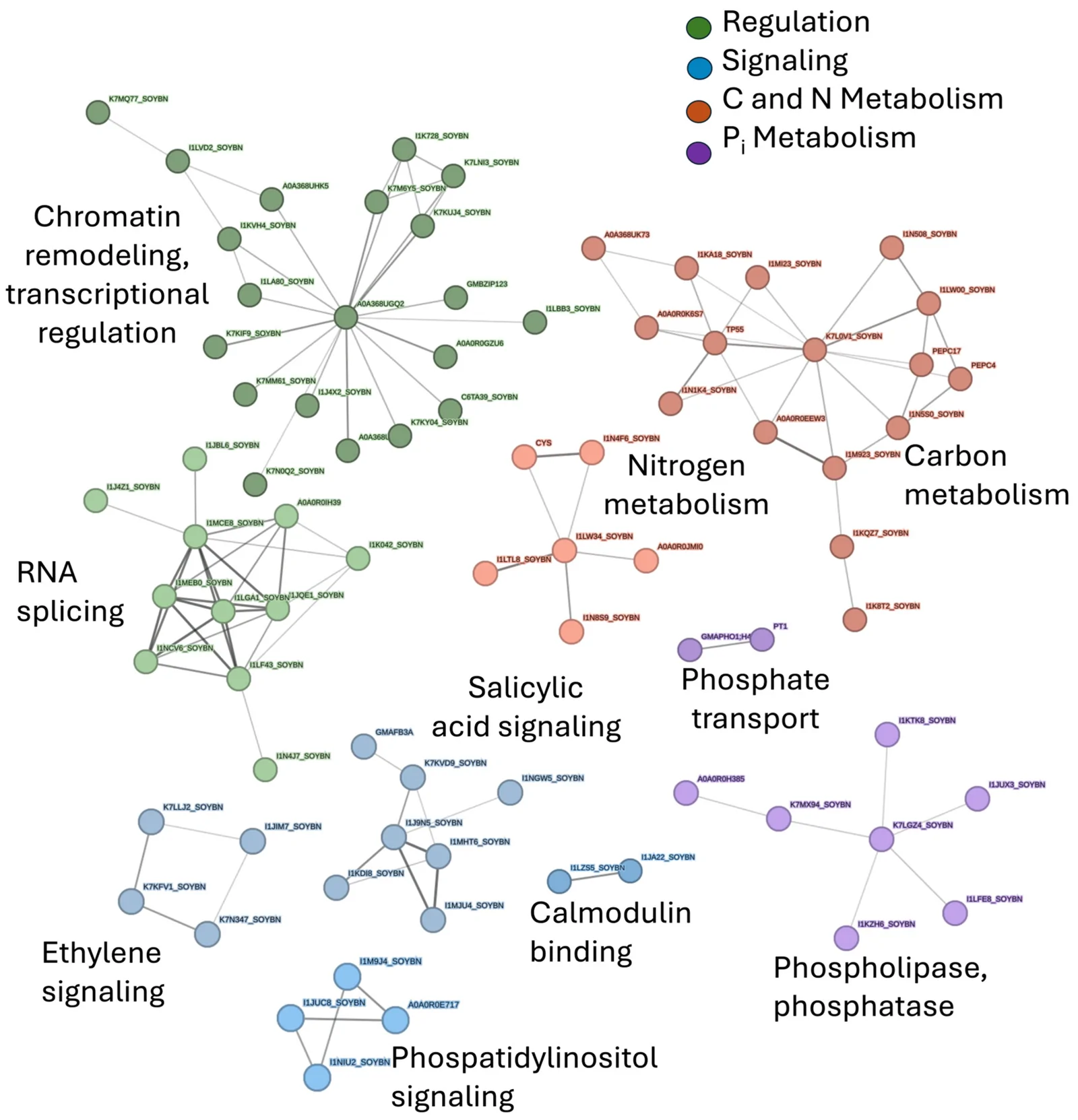
STRING network analysis of genes upregulated by phosphate deficiency under light. All 364 genes upregulated under −P in light were submitted to STRING, and the resulting network was partitioned using the Markov Cluster Algorithm (MCL). Nodes are grouped into broad functional categories: Regulation (green), Signaling (blue), Carbon and Nitrogen Metabolism (brown), and Pi Metabolism (purple). Labels indicate the predominant functions of the displayed clusters based on STRING annotations. All clusters shown correspond to significantly enriched interaction networks, with cluster-specific STRING PPI enrichment *p*-values ranging from <1 × 10^−4^ to <1 × 10^−16^. STRING integrates predicted and experimentally supported interactions from multiple species; therefore, the clusters indicate general functional associations rather than confirmed physical interactions in soybean.

The largest displayed modules were associated with chromatin remodeling, transcriptional regulation, and RNA processing and contained multiple transcription factors and chromatin- or RNA-associated proteins. Additional modules were associated with carbon and nitrogen metabolism and included enzymes involved in glycolytic and amino acid pathways. Other clusters contained proteins linked to salicylic acid, ethylene, phosphatidylinositol, and calcium-related signaling, while a distinct phosphate-metabolism module included phosphate transporters and phosphatases (Figure 7).

### 2.8. Root Sucrose Levels Are Reduced Under Darkness

To investigate one physiological factor that could contribute to the attenuated phosphate-starvation response in darkness, we measured sucrose levels in standardized pooled root samples. Previous studies have shown that shoot-to-root sucrose transport and sucrose signaling promote phosphate-starvation responses [28,29,30]. We therefore asked whether continuous darkness was associated with reduced sucrose accumulation in roots.

**Figure 8:**
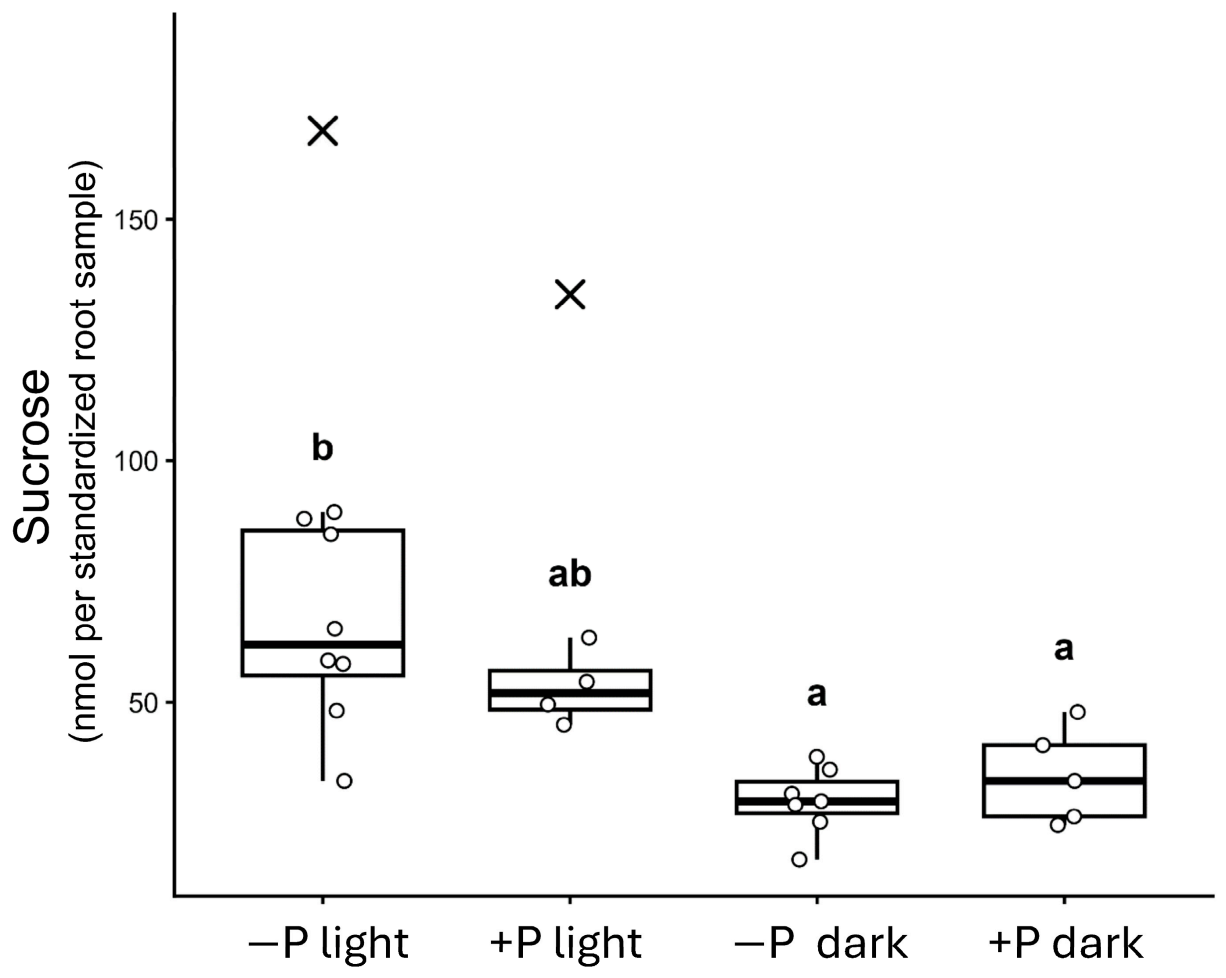
Root sucrose levels under light and dark conditions. Sucrose was quantified in standardized pooled root samples, each consisting of five 4 cm root segments including the root tip. Values are shown for plants exposed for 30 h to +P or −P conditions under a standard light/dark cycle or continuous darkness (*n* = 5–8 biological replicates). Boxplots show the median and interquartile range; open circles represent individual observations, and × symbols indicate flagged outliers. Different letters indicate significant differences among treatment conditions by Tukey’s HSD (*p* < 0.05); flagged outliers were excluded from the statistical analysis.

Root sucrose levels were significantly lower under continuous darkness than under the standard light/dark cycle (two-way ANOVA, light effect, *p* < 0.001; Figure 8). P_i_ availability and the light × P_i_ interaction were not significant. The reduced sucrose levels in dark-grown samples are consistent with limited carbon supply as one potential contributor to the weaker transcriptional response to P_i_ deficiency.

Factorial analysis indicated that illumination had a significant effect on root sucrose content (Type II ANOVA, F(1,20) = 27.92, *p* < 0.001), whereas neither phosphate (F(1,20) = 0.33, *p* = 0.573) nor the phosphate × illumination interaction (F(1,20) = 2.38, *p* = 0.139) was significant. Including the two flagged observations did not change the overall conclusion: illumination remained significant (*p* = 0.001), while phosphate treatment and the phosphate × illumination interaction were nonsignificant.

## 3. Discussion

### 3.1. Under Light, Phosphate Deficiency Activates a Broad Transcriptional Response

P_i_ deficiency triggered a broad transcriptional response in soybean roots under light, including the induction of P_i_ transporters, phosphatases, lipid-remodeling pathways, and transcriptional regulators previously associated with P_i_ deficiency. Genes encoding P_i_ transporters such as *PHT1* and *PHO1* were strongly induced, consistent with their established roles in high-affinity uptake and xylem loading and with the known relief of PHO2-mediated repression under P_i_ limitation [43]. The induction of *PTEN2α* and phospholipases, together with enrichment for lipid metabolism, is consistent with the shift from phospholipids to P-free membrane lipids characteristic of phosphate-starvation responses [22,44].

In total, 364 genes were upregulated under −P in light, spanning transport, signaling, transcriptional regulation, and metabolism. This set likely represents a conservative subset of the broader soybean phosphate-starvation response. For example, O’Rourke et al. reported over 1000 upregulated genes in soybean roots after 24 h of −P_i_ treatment under standard growth conditions [45]. Differences in genotype, treatment duration, sequencing platform, developmental stage, sampling, and statistical analysis likely contributed to the smaller DEG set identified here. Our alternative analysis using LRDE identified 982 genes upregulated by −P under light, a number more similar to what was reported by O’Rourke et al. Independent of the exact number of DEGs, the overlap in well-established P_i_-responsive genes and pathways, including P_i_ transport, phosphatases, and lipid-remodeling processes, indicates that the present dataset captures key components of the early soybean phosphate-starvation response [8,45].

### 3.2. Darkness Attenuates the Response to Phosphate Deficiency

In contrast to the broad response seen under light, P_i_-deficient roots in darkness showed very limited transcriptional activation, with only four genes passing standard significance thresholds. Among these was an L10-interacting MYB-domain transcription factor. In *Arabidopsis*, several MYB proteins function as key components of phosphate-starvation signaling networks [46,47,48,49,50], and L10-interacting MYB factors have been linked to nutrient and ribosomal stress [40].

When statistical stringency was relaxed (|log_2_FC| > 1; padj < 0.1), a total of 13 upregulated genes were identified, including two additional MYB-domain transcription factors: *PHR11*, a homolog of the central phosphate-starvation response regulator *PHR1* [50], and another L10-interacting MYB factor. Both were also identified as significantly upregulated in the LRDE analysis. Stringent fold-change and adjusted *p*-value thresholds can exclude genes with weaker or more variable responses, but even the relaxed criteria identified only a small set of −P-responsive genes in darkness. Thus, the limited response observed in roots from dark-grown plants was not simply a consequence of stringent statistical thresholds.

The strong attenuation of the P_i_-deficiency response in darkness was also supported by an independent differential-expression analysis using LRDE, a method developed for long-read RNA-seq data. Although LRDE identified more DEGs than DESeq2, the contrast between light and darkness remained pronounced, with 982 genes upregulated by P_i_ deficiency under light compared with only 46 in darkness. The pathway-level results were also broadly consistent between methods: MAPK signaling was enriched among light-upregulated genes in both analyses, whereas no KEGG pathway was identified among the 46 LRDE dark-upregulated genes. Together, these results indicate that the strongly reduced P_i_-deficiency response in darkness is robust to the differential-expression method used.

Because local P_i_ responses can involve small cell populations in the root apex and epidermal tissues [38,39], highly localized responses may have been diluted in bulk RNA-seq despite inclusion of the root apex in our samples. The strongly attenuated −P response in darkness is, however, unlikely to reflect a general loss of transcriptional responsiveness. The UpSet analysis showed that thousands of genes were upregulated in response to darkness (Figure 2b), demonstrating the ability of roots from dark-grown plants to induce gene expression. Also, normalized expression values for representative phosphate-starvation response genes showed that these transcripts were readily detectable in darkness (Table 2), but generally failed to increase strongly in response to −P. In addition, the LRDE analysis indicates that darkness does not completely abolish the transcriptional response to P_i_ deficiency, but leaves a much smaller residual response.

Direct comparison of −P-associated fold changes further showed that genes induced under light generally showed attenuated responses in darkness rather than changes in the opposite direction (Figure S2). Interaction-ranked GSEA likewise indicated that this attenuation extended across coordinated signaling, transport, and metabolic pathways. Only 11 genes showed significant light × phosphate interaction effects (Table S2), including five chalcone synthase genes that were induced by −P under light but repressed in darkness. This pattern suggests that (iso)flavonoid metabolism may be especially sensitive to the interaction between light conditions and P_i_ availability.

### 3.3. Reduced Sucrose May Contribute to the Attenuated Response in Darkness

In the present study, root sucrose levels were significantly lower under continuous darkness than under the standard light/dark cycle. This is consistent with previous studies in which dark treatment was used to reduce endogenous sucrose levels [29,51,52,53], including evidence that 24 h of darkness lowers sucrose concentrations in *Arabidopsis* roots [51]. Sucrose-mediated regulation of P_i_-responsive genes has been documented in several plant species, particularly in *Arabidopsis* [28]. Reduced sucrose availability may therefore be one contributor to the attenuated Pi-responsive transcription observed in darkness.

In Arabidopsis, sucrose accumulation is required not only for P_i_-deficiency signaling but also for activation of Fe-deficiency responses, with darkness suppressing *FRO2* and *IRT1* induction by limiting sucrose delivery to roots [51]. Thus, sucrose may serve as a shoot-derived signal in root responses to several nutrient deficiencies.

In soybean, sucrose-feeding experiments similarly showed that exogenous sucrose enhances root growth and induces metabolic signatures resembling P_i_ starvation [54], further supporting a link between sucrose availability and phosphate-starvation responses. However, in white lupin, exogenous sucrose induced cluster-root formation, a characteristic response to P_i_ deficiency, without activating several genes associated with mature cluster-root function, indicating that sucrose alone is not sufficient to reproduce the full functional response to P_i_ deficiency [55].

### 3.4. Light Signaling May Also Contribute to the Attenuated Response in Darkness

Light also affects nutrient-responsive transcription through pathways that are not mediated solely by sucrose [24]. In Arabidopsis, light-signaling regulators including FHY3, FAR1, and HY5 have been shown to regulate *PHR1* expression and phosphate-starvation responses [27]. Shoot light signaling may influence root phosphate-starvation responses through systemic regulators such as HY5 [25], independently of changes in sucrose availability.

In our dataset, numerous genes annotated to light-signaling processes were differentially expressed between light and darkness, including phytochrome- and cryptochrome-related genes, far-red signaling-related components, and several B-box transcriptional regulators. Most of these responses were shared between +P and −P conditions. However, 10 genes met the dark-versus-light differential-expression criteria under only one phosphate condition, and all 10 were upregulated in darkness. Seven of these genes met the criteria only under −P and included *PHYA*, *PHYB*, a phytochrome B-2-like gene, and a cryptochrome-2-like gene. However, none of the 51 light-signaling genes showed a significant light × phosphate interaction after multiple-testing correction.

Light-signaling genes showed little direct transcriptional response to P_i_ deficiency itself. Only one of the 51 genes, LOC100808627, encoding a cryptochrome-2-like protein, was significantly induced by −P under light, and none was significantly responsive to −P in darkness. Together, these results indicate that darkness strongly alters light-signaling gene expression in soybean roots, but do not identify a specific transcriptional light-signaling component that explains the attenuation of the Pi-deficiency response.

Direct illumination of roots can strongly alter phosphate-starvation responses and root transcriptional programs in Arabidopsis [56]. In the present study, roots were maintained in darkness in all treatments, minimizing direct root photoreception and focusing the comparison on systemic consequences of shoot light exposure.

**Figure 9:**
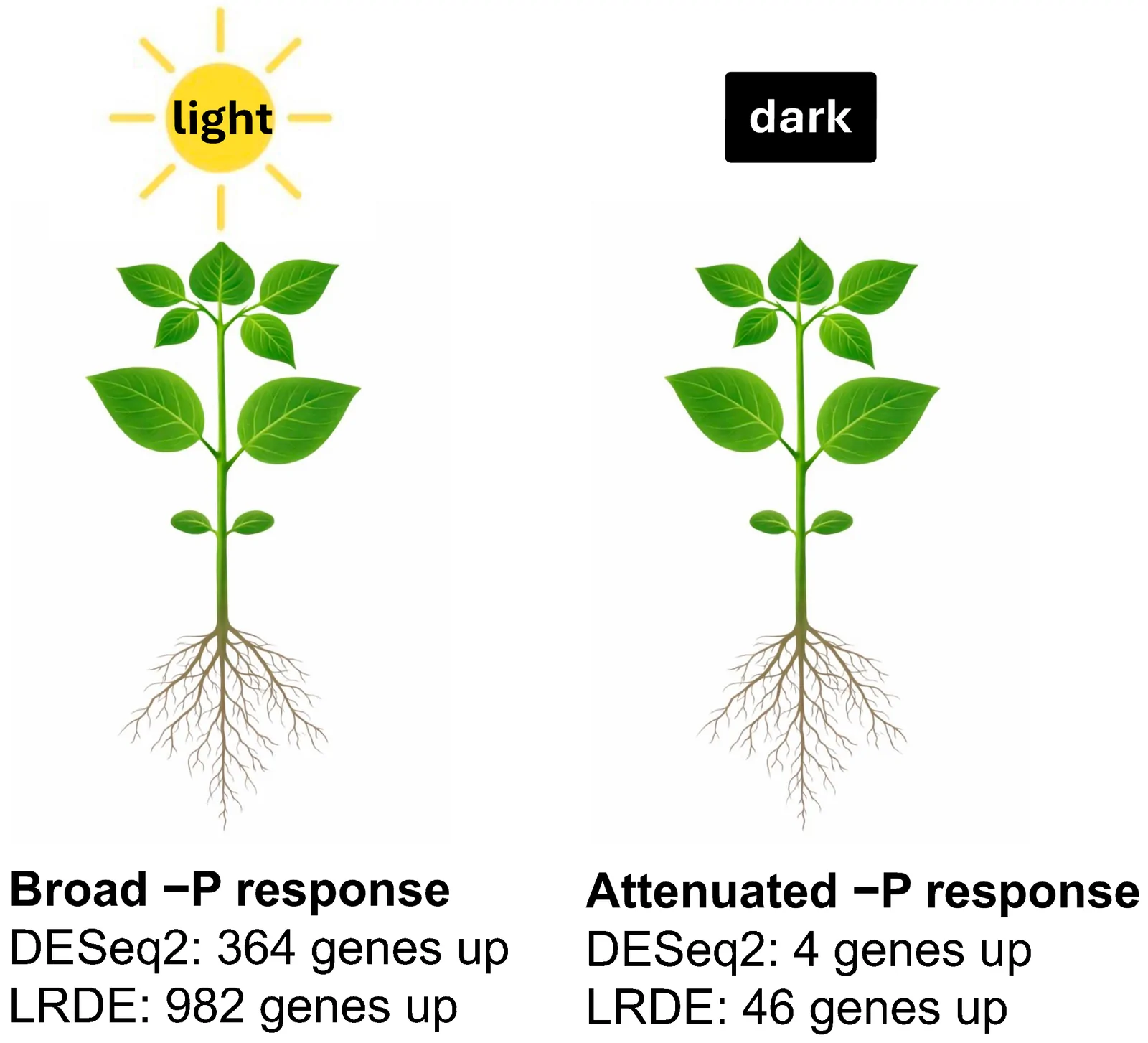
Schematic summary of the phosphate-starvation response under light and darkness in soybean roots. Under light conditions, P_i_ deficiency induced a broad transcriptional response, including transcription factors, P_i_ transporters, lipid-remodeling genes, signaling components, and metabolic genes. In darkness, root sucrose levels were reduced and the transcriptional response to P_i_ deficiency was strongly attenuated, with only four genes significantly upregulated in the DESeq2 analysis. Reduced root sucrose and altered light-signaling gene expression are discussed as possible contributors to this attenuation.

Together, these findings support a model in which darkness strongly attenuates the broad phosphate-starvation response observed under light, with reduced root sucrose and altered light signaling as possible contributing factors (Figure 9).

### 3.5. Limitations and Future Directions

Several intriguing possibilities could explain how darkness attenuates the phosphate-starvation response, including direct effects of light-signaling pathways, indirect effects mediated through reduced carbon availability, or a combination of both. Darkness affects numerous physiological and regulatory processes, including circadian, metabolic, hormonal, and oxidative pathways [57,58], and also reduces carbon availability in roots. Darkness may reduce growth and P_i_ demand, which could contribute to or cause the weaker phosphate-starvation response. In this study, Pi deficiency refers to the experimental withdrawal of P_i_ from the nutrient solution to examine an early response, rather than to a defined degree of tissue P_i_ depletion.

Future experiments combining darkness with sucrose supplementation, or selectively disrupting shoot-to-root carbon transport under light, could help distinguish carbon-dependent from other effects of darkness and test whether restoring sucrose availability rescues the broad phosphate-starvation response in darkness. In parallel, experiments varying light intensity and spectral quality could further separate effects of photosynthetic carbon supply from light-signaling pathways and clarify how specific light conditions modulate the root response to P_i_ deficiency.

The 30 h treatment captured an early transcriptional response, and additional P_i_-responsive genes may emerge at other time points. Because only a single time point was examined, we cannot determine whether darkness reduces the initial induction of the phosphate-starvation response or alters its timing or duration. In addition, although sampling included approximately 4 cm of the apical root region, localized or low-abundance responses may have been diluted in bulk RNA-seq. Higher-resolution approaches, including cell type-resolved or spatial transcriptomics, could help identify P_i_-responsive cell populations that remain active in darkness.

The study nevertheless shows a clear contrast between the extensive P_i_-responsive transcriptome under light and the small P_i_-responsive subset detected in darkness. Determining how light and carbon availability regulate these responses could help identify conditions under which P_i_ acquisition is constrained by carbon supply.

## 4. Materials and Methods

### 4.1. Overview

This study investigated transcriptomic responses of soybean (*Glycine max*) roots to phosphate (P_i_) availability under light and dark conditions using Oxford Nanopore (Oxford Nanopore Technologies, Oxford, UK) long-read sequencing. Plants were grown hydroponically under controlled conditions and exposed to P_i_ withdrawal for 30 h under a standard light/dark cycle or continuous darkness. Each of the four independent hydroponic experiments constituted one experimental block and included all four treatment combinations (+P light, −P light, +P dark, and −P dark), with one plant sampled per treatment in each block. Root RNA was extracted for full-length cDNA library preparation, sequencing was performed on MinION flow cells, and reads were processed through a reproducible bioinformatics workflow.

### 4.2. Plant Growth and Treatment

*Glycine max* cv. Williams 82 seeds, kindly provided by John Harada and Julie Marie Pelletier (University of California, Davis), were germinated for 3–4 days and transferred to hydroponic containers containing 8 L of half-strength Hoagland solution. The phosphate-sufficient (+P) solution contained 0.5 mM CaHPO_4_ as the phosphate source. For −P treatments, phosphate was withdrawn by omitting CaHPO_4_ and replacing it with CaSO_4_ to maintain equivalent calcium availability. CaHPO_4_ was selected because it maintained the nutrient solution near pH 5.8 and allowed calcium concentrations to be balanced between +P and −P treatments through CaSO_4_ supplementation. Under these growth conditions, CaHPO_4_ dissolved readily, and no visible P_i_ precipitate was observed. The nutrient solution was replenished every 3 days.

Plants were grown in a temperature-controlled chamber maintained at 27 °C under a 12 h light/12 h dark photoperiod, with lights on at 6:00 a.m. and lights off at 6:00 p.m. After 10 days, cotyledons were removed to reduce stored P_i_ reserves. At 14 days, plants were exposed for 30 h to one of four conditions: +P or −P under the standard light/dark cycle, or +P or −P under continuous darkness. Darkness was imposed by turning off the lights on the shelf assigned to the dark treatment, while all plants remained in the same temperature-controlled growth chamber. P_i_ and darkness treatments began at 6:00 a.m., at the onset of the light period. Samples were harvested 30 h later at 12:00 p.m. the following day, corresponding to 6 h into the second light period for plants maintained under the standard light/dark cycle.

For RNA-seq, each treatment was repeated in four independent hydroponic experiments. Each biological replicate consisted of roots from a single plant grown under the same controlled growth-chamber conditions. At harvest, ~4 cm segments of the main root axes, including the root tip, were cut off, immediately flash-frozen in liquid nitrogen, and stored at –80 °C until RNA extraction.

### 4.3. RNA Isolation and Quality Assessment

Total RNA was isolated from frozen root tissue using the RNeasy Plant Mini Kit (Qiagen, Hilden, Germany) according to the manufacturer’s protocol for “Purification of Total RNA from Plant Cells and Tissues, and Filamentous Fungi.” RNA concentration and integrity were evaluated using a Qubit 4 Fluorometer (Thermo Fisher Scientific, Waltham, MA, USA) with the Qubit RNA High Sensitivity and RNA IQ assays. The RNA IQ assay measures the proportion of large or structured RNA relative to small RNA to assess sample integrity. Only samples with RNA IQ > 7 were used for sequencing; most samples had RNA IQ > 8.

### 4.4. cDNA Library Preparation and Quantification

For each sample, 500 ng of total RNA was used to synthesize cDNA using the cDNA-PCR Sequencing Kit V14 with Barcoding (Oxford Nanopore Technologies, Oxford, UK), following the manufacturer’s instructions. cDNA library concentration and quality were verified with an Agilent TapeStation (Agilent Technologies, Santa Clara, CA, USA). For each independent hydroponic experiment, the four barcoded cDNA libraries were pooled to a final amount of 50 fmol.

### 4.5. Nanopore Sequencing

Sequencing was performed using the Oxford Nanopore Technologies cDNA-PCR Sequencing V14—Barcoding (SQK-PCB114.24) protocol with R10.4.1 flow cells (FLO-MIN114). Flow cells were primed and loaded with pooled cDNA according to the manufacturer’s instructions. Each MinION sequencing run was conducted for 72 h. For each independent hydroponic experiment, all four treatment libraries were included on both flow cells. Reads from the two flow cells were demultiplexed separately, and then combined by sample before alignment and gene-level counting.

### 4.6. Nanopore Read Processing, Alignment, and Quantification

Raw reads were processed in MinKNOW v 25.05.14 (Oxford Nanopore Technologies), retaining only passed reads (Q > 7) and demultiplexed during sequencing. MinKNOW basecalling was used consistently for all downstream analyses. Sequencing data were transferred to the Expanse high-performance computing cluster (San Diego Supercomputer Center, USA).

**Read orientation and trimming:** Full-length cDNA reads were identified and oriented using *Pychopper v2.7* (RRID:SCR_018966; https://github.com/epi2me-labs/pychopper (accessed on 28 July 2026)) with the -m edlib setting.

**Read alignment:** Reads were aligned to the *G. max* reference genome (GCF_000004515.6 v4.0; downloaded 1 Oct 2025) using *Minimap2 v2.30-r1287* [59]. Alignments were sorted and indexed with *Samtools v1.20* [60].

**Gene quantification:** Gene counts were obtained using *featureCounts v2.0.6* [61] in long-read mode (-L) with single-end, stranded settings (-s 1); multi-mapped reads were not counted (-M was not used). The resulting count matrix and summary tables were exported for downstream analysis in R.

**Quality control:** Mapping statistics were evaluated using *samtools flagstat* and *featureCounts* summary reports.

### 4.7. Differential Expression and Functional Analyses

**DESeq2:** Differential expression analyses were performed in R (v4.4.0). Raw count matrices from *featureCounts* were imported into the DESeq2 (v1.42.0) package [62] for normalization and differential expression analysis. DESeq2 normalization accounts for differences in library size and overall expression levels using a median-of-ratios approach, preventing these differences from biasing differential expression estimates. Gene-level differential expression was analyzed using DESeq2, which models integer count data generated here from long-read alignments assigned to annotated genes. The present analysis focused on gene-level transcriptional responses rather than transcript- or isoform-level variation. Before differential expression analysis, genes were prefiltered by requiring at least 10 raw counts in at least 3 biological replicates within any one of the four treatment groups. This removed low-abundance genes prior to DESeq2 dispersion estimation. DESeq2 size-factor-normalized counts were used to summarize expression levels in Table 2. Variance-stabilizing transformation (VST) was applied for PCA and heatmap visualization. Genes with adjusted *p* < 0.05 and |log_2_ fold change| > 1 were considered significantly differentially expressed. Genes shown in Table 2 were selected because of their established roles in P_i_ uptake, transport, or transcriptional signaling.

**LRDE:** As an independent analysis developed for long-read RNA-seq data, differential expression was also assessed using LRDE (v0.99.6) [42] in R (v4.6.1). The LRDE analysis used the same raw gene-level count matrix and experimental metadata as the DESeq2 analysis. Genes were first filtered using the same criterion applied in the DESeq2 analysis: at least 10 raw counts in at least three biological replicates within at least one of the four treatment groups. Light and dark samples were then analyzed separately for the −P versus +P comparison. Because LRDE dispersion estimation failed for genes with zero counts in all replicates of one phosphate treatment group, genes completely absent from either the +P or −P group within each light condition were excluded before model fitting; these genes showed only sparse expression in the opposing treatment group. Differential expression was assessed using the LRDE hurdle likelihood-ratio test, and *p*-values were adjusted for multiple testing using the Benjamini–Hochberg method. Genes were considered differentially expressed at adjusted *p* < 0.05 and |log_2_FC| > 1. For the LRDE analysis, log_2_FC was calculated from mean size-factor-normalized counts for −P relative to +P after adding a pseudocount of 1.

**Principal component analysis (PCA)** was performed on variance-stabilized (VST) counts using DESeq2. The PCA was calculated using the top 500 most variable genes, and the first two principal components were visualized with *ggplot2* v4.0.0. Replicate identifiers were added to the plot to assess clustering within and across treatments.

**UpSet plots:** Overlaps among DEG sets were visualized using UpSetR v1.4.0 [63] with separate plots for up- and downregulated genes.

**Volcano plots** were generated in R from DESeq2 differential expression results using adjusted *p* < 0.05 and |log_2_FC| > 1 thresholds.

**Heatmaps:** Expression patterns were visualized using ComplexHeatmap v2.20.0 [64], with per-gene *z*-score scaling of VST counts. Hierarchical clustering was performed using Euclidean distance and complete linkage.

**Fold-change comparison and interaction analysis:** Pairwise differential-expression analyses were performed within each light or phosphate condition using DESeq2 models that included experimental replicate as a blocking factor (~ replicate + P or ~ replicate + light, respectively). For genes significantly induced by −P under light, log_2_ fold changes for −P versus +P were compared between light and dark conditions. Light × phosphate interactions were tested using the model ~ replicate + light * P, with light and +P as the reference levels. The interaction coefficient therefore represents the difference in the phosphate response between darkness and light. Genes with an adjusted interaction *p* < 0.05 were considered significant, without an additional fold-change threshold.

**KEGG enrichment**: KEGG pathway analysis was performed using clusterProfiler v4.10.0 [65], with the soybean organism code *gmx*. Gene identifiers were mapped to Entrez gene IDs, and enrichment was tested separately for each DEG set using enrichKEGG with keyType = “ncbi-geneid” and a minimum gene-set size of five. The background consisted of genes that passed the expression filtering and had a valid Entrez ID. Benjamini–Hochberg correction was applied for multiple testing. Pathways were retained for reporting when adjusted *p* < 0.1 and at least three DEGs mapped to the pathway. Up to 20 pathways were displayed per comparison and direction.

**Gene set enrichment analysis (GSEA):** Preranked KEGG gene set enrichment analysis was performed with gseKEGG in clusterProfiler using the soybean organism code gmx, Entrez gene identifiers, and the signed DESeq2 Wald statistic for the light × phosphate interaction as the ranking metric. Genes lacking an Entrez identifier or a valid Wald statistic were excluded. When multiple genes mapped to the same Entrez identifier, the gene with the largest absolute Wald statistic was retained. Pathways containing at least 10 mapped genes were tested, and *p*-values were adjusted using the Benjamini–Hochberg method. For visualization, clearly non-plant pathway labels were excluded, and the 20 remaining pathways with the smallest adjusted *p*-values were displayed.

**Transcription factor families**: Transcription factors were identified by cross-referencing −P-responsive genes with a curated soybean transcription factor list from PlantTFDB [66], and transcription factor families were summarized descriptively. Overrepresentation of transcription factors among −P-responsive genes was tested using Fisher’s exact test with all expressed genes as background. No significant enrichment was detected.

**GO enrichment:** Gene Ontology (GO) enrichment was performed with *g:Profiler* (organism = “gmax”) separately for up- and downregulated DEG sets using an FDR threshold of < 0.05. The background consisted of all expressed genes. Results were summarized using Biological Process terms, with closely related terms collapsed for visualization.

**Light-signaling gene analysis:** To examine light-signaling components directly, soybean genes were annotated using NCBI Gene Ontology annotations (gene2go, *Glycine max* taxon ID 3847, accessed 25 August 2026) [67]. Genes annotated to Biological Process terms related to photomorphogenesis, light signaling or phototransduction, and responses to red, far-red, or blue light were considered light-signaling genes. Representation of established light-signaling components was additionally checked using NCBI Gene annotations and NCBI BLAST web searches. Of these, 51 genes were retained after the low-abundance expression filter used for the differential-expression analysis. Their differential expression was examined for dark versus light under +P and −P conditions and for −P versus +P within each light condition using the same DESeq2 significance criteria applied in the main analysis. Light × phosphate interaction effects were evaluated using the DESeq2 interaction model described above.

**STRING network analysis:** Protein–protein interaction networks were analyzed using STRING v12.0 [68] with *Glycine max* as the reference organism. All genes upregulated by −P under light were submitted together, and densely connected subnetworks were identified using the Markov Cluster Algorithm with the default inflation parameter of 3. Cluster membership was exported from STRING, and cluster labels used for visualization were based on STRING Gene Ontology enrichment results.

### 4.8. Sucrose Quantification

Sucrose levels were quantified from root samples collected under the same treatment conditions used for transcriptomic analysis, with five to eight biological replicates per treatment. For each biological replicate, five ~4 cm root segments, including the root tip, were harvested, pooled, immediately flash-frozen in liquid nitrogen, and stored at −80 °C. Samples were lyophilized in a freeze dryer (Labconco FreeZone 2.5, Kansas City, MO, USA) and then ground into a fine powder with a pestle.

Sucrose content was determined using a fluorometric Sucrose Assay Kit (Novus Biologicals™, Centennial, CO, USA) according to the manufacturer’s instructions. Due to low biomass, sucrose values are reported as total sucrose per standardized pooled root sample. Sucrose data were analyzed using a two-way ANOVA with light condition (light vs. dark) and P_i_ availability as factors, followed by Tukey’s HSD pairwise comparisons.

## 5. Conclusions

Under light conditions, soybean roots showed a broad phosphate-starvation response. In darkness, the response to P_i_ deficiency was strongly attenuated, although dark-grown samples remained transcriptionally responsive, as shown by the upregulation of thousands of genes in response to darkness itself. Most genes induced by P_i_ deficiency under light showed attenuation rather than reversal in darkness, with only a few becoming downregulated. Root sucrose levels were lower in darkness, and numerous light-signaling genes were differentially expressed between light and darkness. Although the transcriptomic data did not identify a specific light-signaling component that accounted for the attenuated P_i_ response, reduced carbon availability and altered light signaling remain possible contributors.

## Figures and Tables

**Table 1:** Summary of Oxford Nanopore cDNA sequencing and read-mapping metrics.

Condition	Passed Reads (×10^6^)	Average Read Quality (Q)	N50 (bp)	Mapped Reads (%)
+P light (*n* = 4)	6.9 ± 2.5	11 ± 0	1034 ± 75	96.9 ± 0.5
−P light (*n* = 4)	4.8 ± 1.9	10 ± 0	814 ± 96	93.4 ± 2.6
+P dark (*n* = 4)	6.7 ± 2.2	10 ± 0	879 ± 113	95.6 ± 1.5
−P dark (*n* = 4)	4.9 ± 1.1	11 ± 0	972 ± 103	96.8 ± 0.4

Values are means ± SD of four biological replicates.

**Table 2:** Normalized expressions of phosphate-starvation response genes induced under light. Mean DESeq2-normalized counts from four biological replicates are shown for representative phosphate-starvation response markers under light and dark conditions.

Representative Phosphate- Starvation Markers *	Gene ID	Light (+P)	Light (−P)	−P/+P Light log_2_FC (padj)	Dark (+P)	Dark (−P)	−P/+P Dark log_2_FC (padj)
*PHO1*	PHO1-H4	28.1	68.7	1.5 (0.027)	23.6	31.7	0.43 (0.9)
*PHT1*	PHT1;14	537.5	1111.0	1.0 (0.016)	943.6	806.5	−0.13 (0.9)
*PTEN2α-1*	LOC100799214	38.6	84.0	1.2 (0.012)	111.8	97.5	−0.18 (0.9)
*PTEN2α-2*	LOC100784003	76.9	155.0	1.1 (0.013)	172.2	184.9	0.14 (0.9)
*SAP*	LOC10078702	8.9	24.3	1.5 (0.021)	53.9	71.4	0.42 (0.8)

* *PHO1*, phosphate exporter involved in xylem loading; *PHT1*, high-affinity phosphate transporter; *PTEN2α-1* and *PTEN2α-2*, paralog phosphatidylinositol triphosphate phosphatases; *SAP*, secreted acid phosphatase.

**Table 3:** Upregulated genes in the −P/+P comparison under darkness. Four genes passed standard significance criteria (log_2_FC > 1, adjusted *p* < 0.05) and are listed first, followed by nine additional candidate genes identified using relaxed thresholds (log_2_FC > 1, adjusted *p* < 0.1) to examine whether additional −P-responsive genes could be detected in darkness.

Gene_ID	Description	−P/+P Dark log2FC	−P/+P Dark padj	−P/+P Light log2FC	−P/+P Light padj
*SWEET5*	sugar efflux transporter SWEET5	4.1	0.045	−0.9	0.215
LOC100787217	glucose-6-phosphate 1-epimerase	2.5	0.013	−0.4	0.612
LOC100820117 *	L10-interacting MYB domain- containing protein	2.5	0.019	0.1	NA
LOC100807179 *	uncharacterized LOC100807179	1.2	0.029	−0.7	0.072
LOC100818214 *	WEB family protein	2.7	0.061	0.6	NA
LOC100776105	arabinogalactan protein 9	2.0	0.091	−0.7	0.351
*PHR11* *	homolog to PHR1	1.8	0.061	−0.2	0.738
LOC100775446	aspartyl protease AED3	1.7	0.055	−0.1	0.878
LOC113000488 *	L10-interacting MYB domain- containing protein	1.5	0.077	0.6	NA
LOC100808302	cyclin-dependent kinase B2-2	1.5	0.078	−0.6	0.352
LOC100785581	kinesin-like protein KIN-14U	1.5	0.089	1.7	0.006
LOC100804267 *	uncharacterized	1.5	0.091	0.2	0.766
LOC100802287 *	glucan endo-1,3-beta- glucosidase 7	1.3	0.077	−0.3	0.588

* Also identified as upregulated in the −P/+P comparison under darkness by LRDE analysis. NA: an adjusted *p*-value was not assigned by DESeq2.

**Table 4:** Comparison of DESeq2 and LRDE results for −P versus +P.

Comparison	DESeq2	LRDE	Shared	Overlap (DESeq2 in LRDE)
−P vs. +P, light, up	364	982	337	93%
−P vs. +P, light, down	655	1259	566	86%
−P vs. +P, dark, up	4 (13 *)	46	2 (7 *)	50% (54% *)
−P vs. +P, dark, down	83	119	35	42%

* DESeq2 relaxed criterion: adjusted *p* < 0.1; log2FC > 1.

**Table 5:** Light-signaling genes differentially expressed between dark and light under only one phosphate condition.

Gene Symbol/ID	Description	Dark vs. Light, +P log2FC	Dark vs. Light, +P padj	Dark vs. Light, –P log2FC	Dark vs. Light, –P padj	Light × Phosphate Interaction padj
**Significant dark vs. light under +P only**						
LOC100794842	B-box zinc finger protein 21	1.37	0.004	0.04	0.950	0.466
LOC100790232	B-box zinc finger protein 20	1.86	0.037	1.58	0.077	NA
LOC100787535	uncharacterized	1.06	0.001	0.64	0.056	0.738
**Significant dark vs. light under –P only**						
LOC100780975	BTB/POZ domain-containing protein POB1	−0.20	0.809	2.13	0.008	NA
*PHYA*	phytochrome A	0.93	0.044	1.75	1.05 × 10^−4^	0.635
*PHYB*	phytochrome B-like	0.79	0.208	1.71	0.009	0.693
LOC100794865	phytochrome B-2-like	−0.03	0.969	1.41	0.023	NA
LOC100795495	F-box protein	1.10	0.097	2.21	0.006	NA
LOC100808627	cryptochrome-2-like	0.59	0.109	1.21	5.05 × 10^−4^	0.638
LOC100801613	uncharacterized	0.88	0.001	1.11	4.37 × 10^−5^	0.847

Genes were classified according to the phosphate condition in which they met the differential-expression criteria for dark versus light (adjusted *p* < 0.05 and |log_2_FC| > 1). None of these genes showed a significant light × phosphate interaction after multiple-testing correction.

## Data Availability

Nanopore sequencing data have been deposited in the NCBI Gene Expression Omnibus (GEO) under accession number GSE312797. Additional data supporting this study are available from the corresponding author upon reasonable request.

## Supplementary Materials

The following supporting information can be downloaded at: https://www.mdpi.com/article/10.3390/ijms27177963/s1.

## Abbreviations

The following abbreviations are used in this manuscript:

DEG	differentially expressed genes
GO	Gene Ontology
KEGG	Kyoto Encyclopedia of Genes and Genomes
padj	adjusted *p*-value (Benjamini–Hochberg correction)
P_i_	inorganic phosphate
PCA	principal component analysis
PP_i_	pyrophosphate
RNA-seq	RNA sequencing
