# Supplementary material for: Darkness Attenuates the Early Transcriptional Response to Phosphate Deficiency in Soybean Roots
Source: Int J Mol Sci. 2026 Sep 7;27(17):7963. doi: 10.3390/ijms27177963 (PMC13565979; doi:10.3390/ijms27177963)
Supplement: Supplementary file 1 [file ijms-27-07963-s001.zip › Supplementary_Figure_S1-S5.pdf]

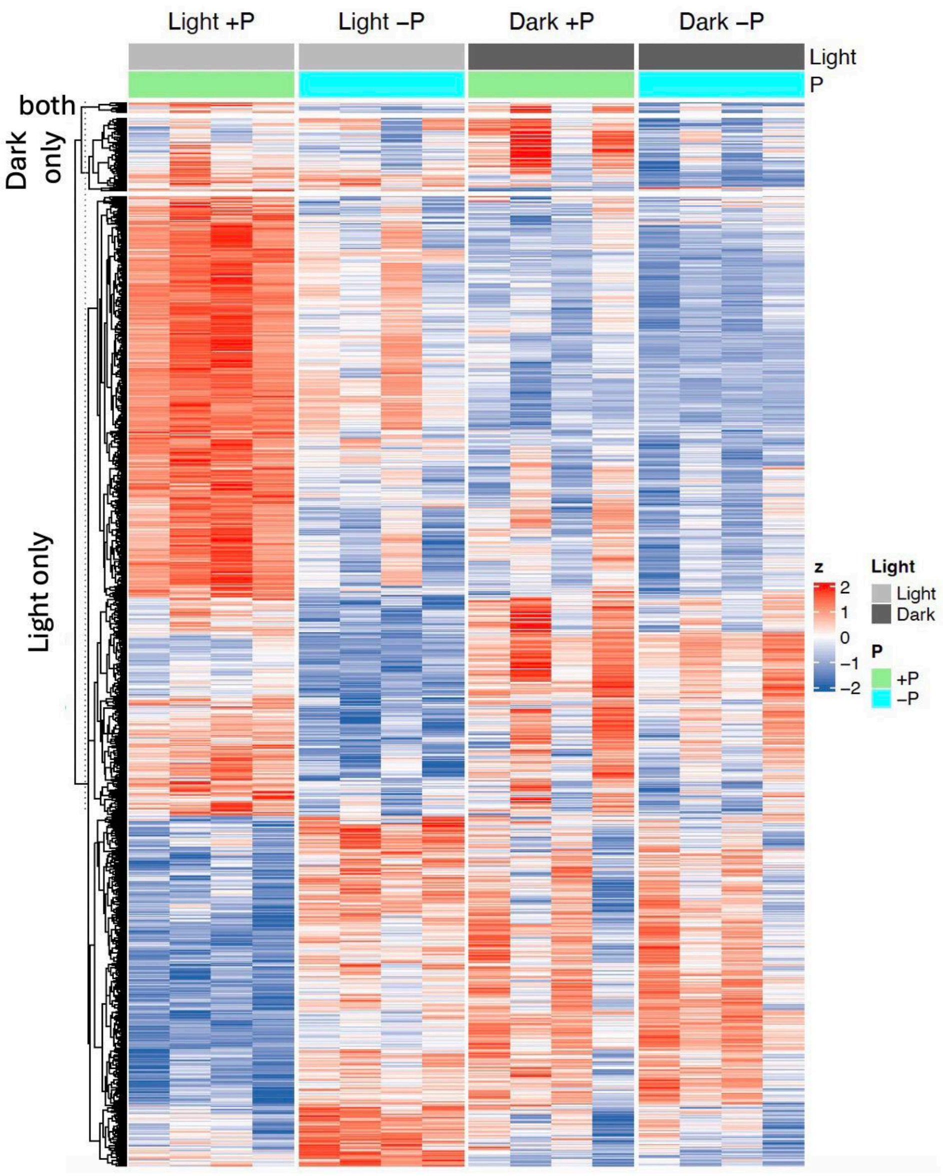

Supplementary Figure S1. Heatmap of genes differentially expressed under P_i deficiency in light and dark conditions. The heatmap includes the combined set of genes differentially expressed in the −P versus +P comparison under light or dark conditions (adjusted $p < 0.05$, $|\log_2 FC| > 1$). Rows represent individual genes, and columns represent biological replicates. Expression values were z-score normalized across samples, with blue and red indicating lower and higher relative expression within each gene, respectively. Column annotations indicate light condition and phosphate status. Row groups indicate genes that were differentially expressed only under light, only in darkness, or under both conditions. The heatmap shows extensive −P-associated transcriptional reprogramming in light-grown roots, whereas comparatively few genes respond to −P in darkness.

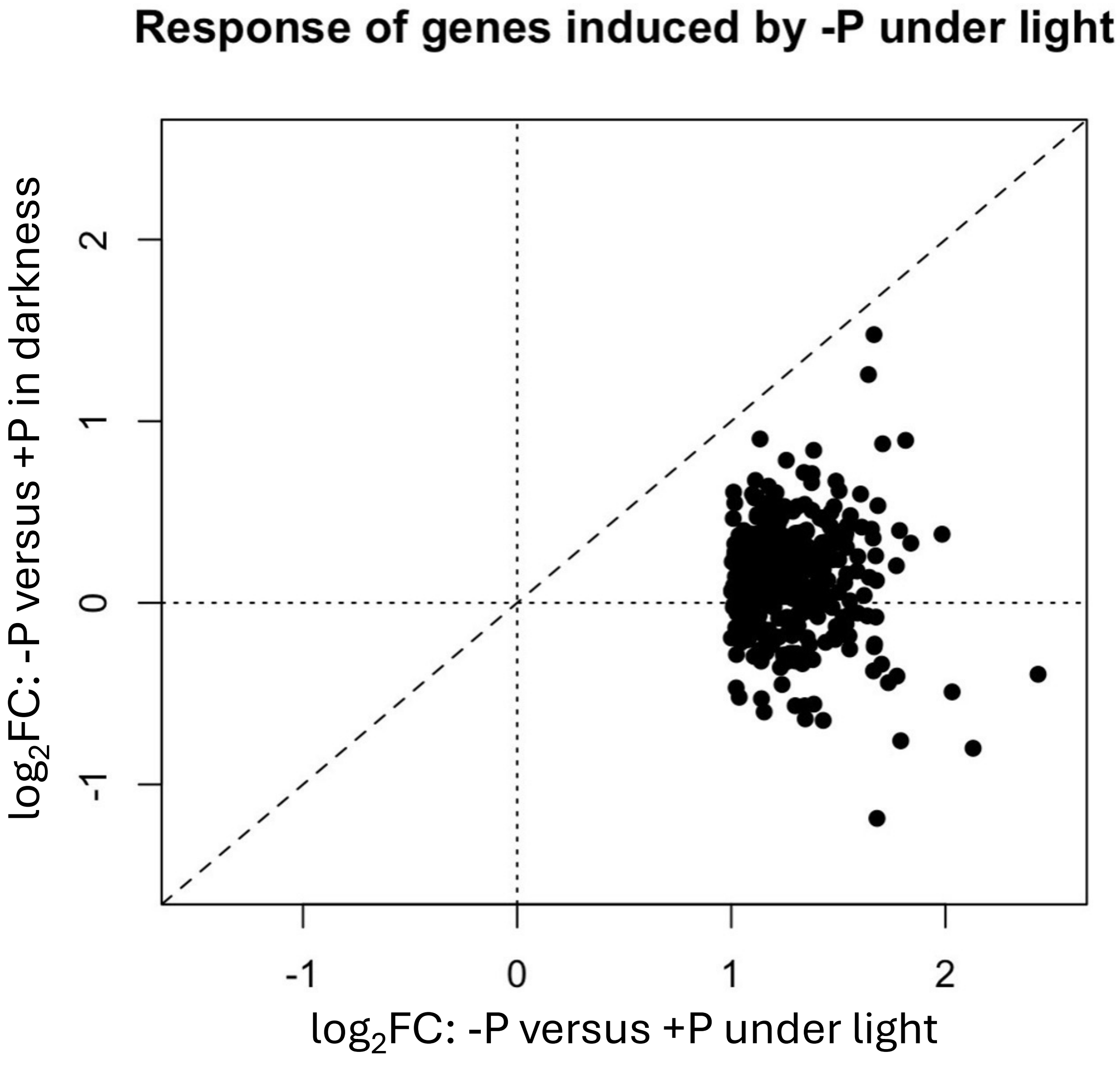

Supplementary Figure S2. Comparison of phosphate-deficiency responses under light and dark conditions. The scatterplot shows $\log_2$ fold changes for −P versus +P under light and darkness for genes significantly induced by −P under light (*padj* < 0.05, $\log_2FC > 1$). The dashed diagonal indicates equal responses under light and darkness, and dotted horizontal and vertical lines indicate no change. Most genes were located well below the diagonal and close to zero on the dark-response axis, indicating that genes induced by −P under light generally showed little response in darkness.

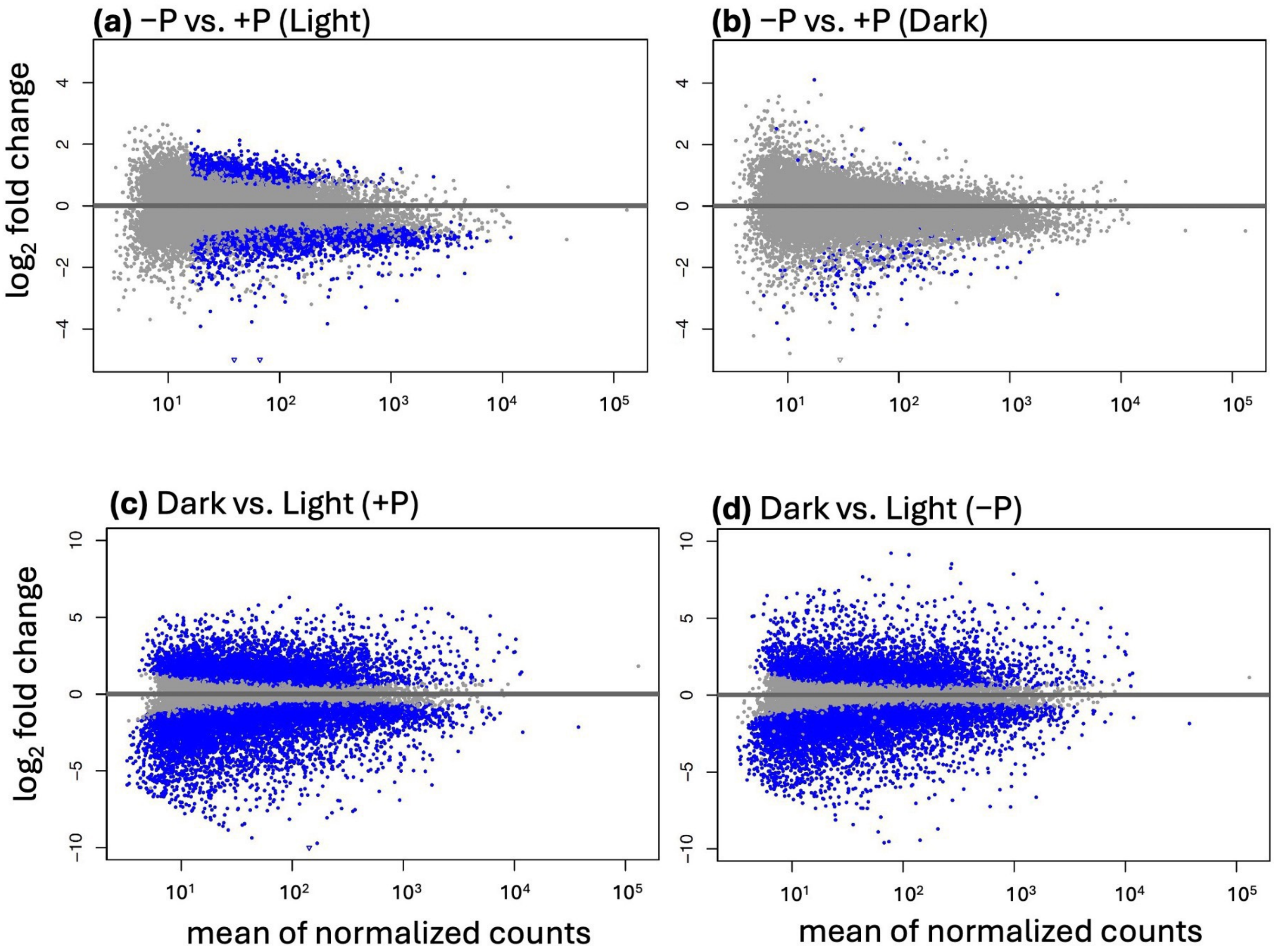

Supplementary Figure S3. MA plots for the four treatment comparisons. Log_2 fold change is plotted against the mean of normalized counts for (a) −P versus +P under light, (b) −P versus +P under darkness, (c) dark versus light under +P, and (d) dark versus light under −P. Blue points indicate genes meeting the relaxed criteria of $|log_2FC| > 1$ and adjusted $p < 0.1$; gray points did not meet these criteria. Triangles indicate genes with log_2 fold changes outside the displayed y-axis range. The sparse distribution of significant genes in panel (b) shows that the limited −P response in darkness remained evident under the relaxed threshold.

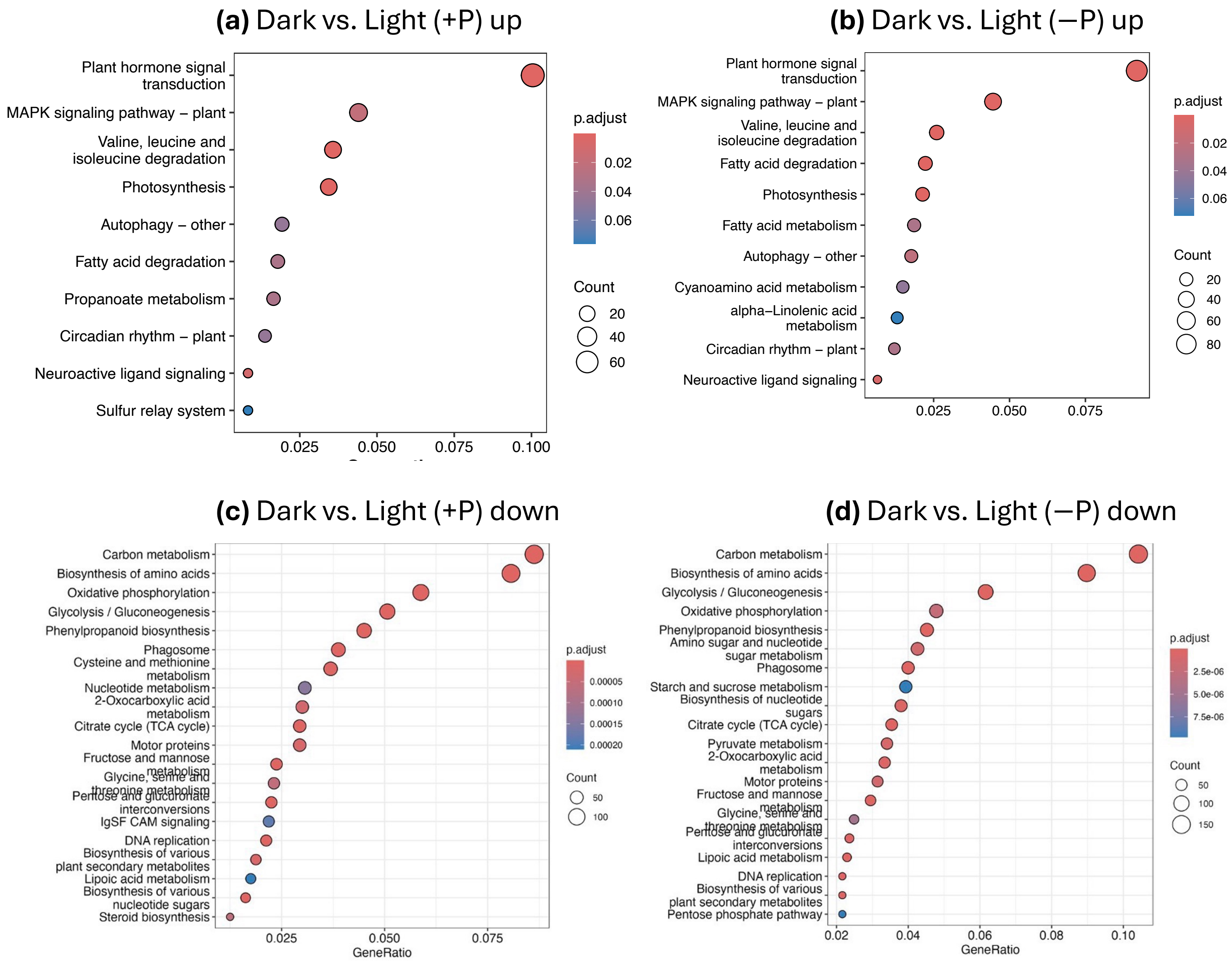

Supplementary Figure S4. KEGG pathway enrichment analyses for dark versus light comparison under phosphate-sufficient (+P) and phosphate-deficient (–P) conditions.

(a, b) KEGG pathways enriched among genes upregulated under dark versus light conditions within +P (4668 genes) or –P (3751 genes) treatments.

(c, d) KEGG pathways enriched among genes downregulated under dark versus light conditions within +P (5712 genes) or –P (5127 genes) treatments.

Circle size represents the number of genes annotated to each pathway, and color indicates the adjusted p-value.

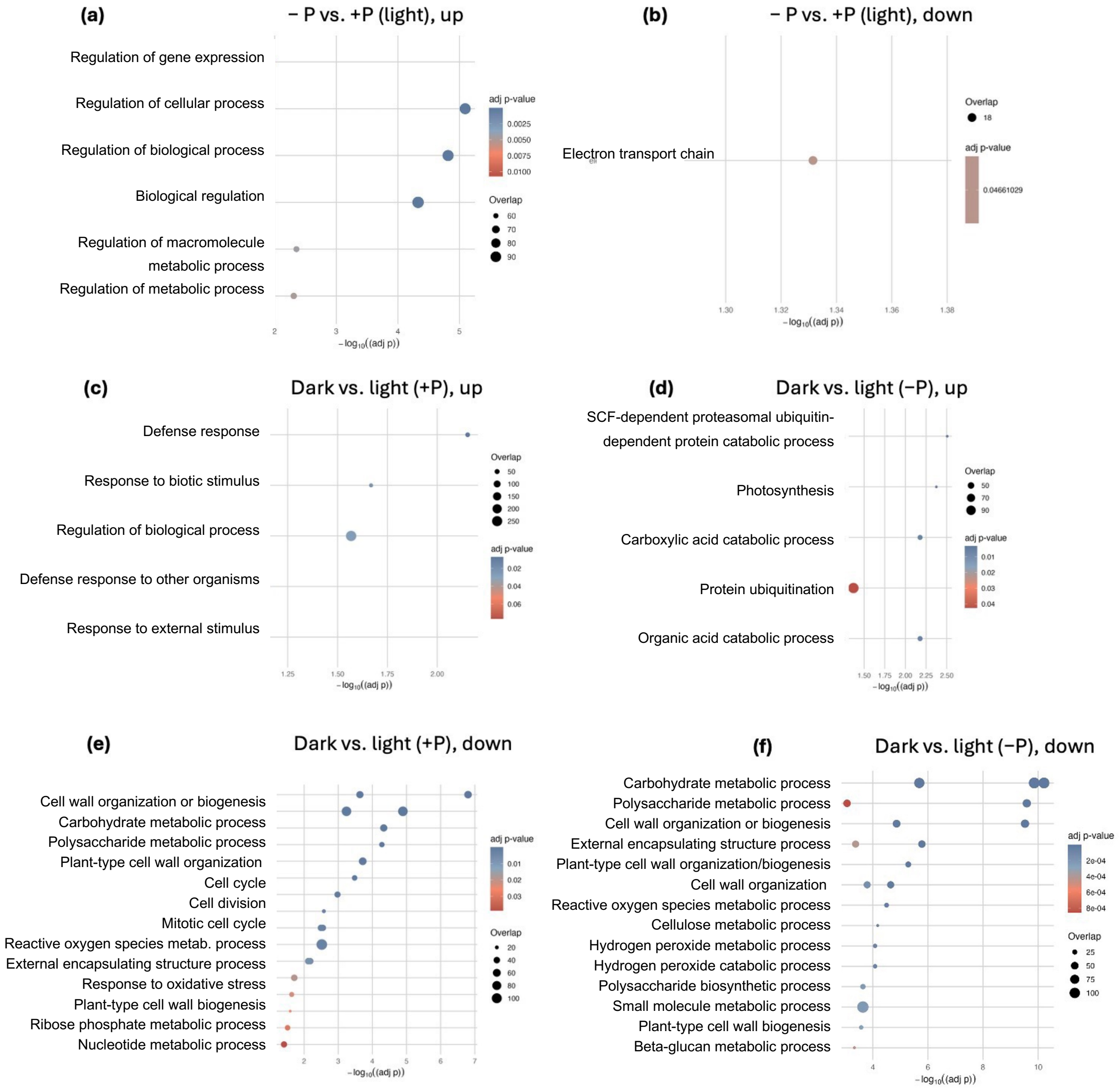

Supplementary Figure S5. GO Biological Process enrichment of differentially expressed genes under phosphate deficiency (–P) and phosphate sufficiency (+P) in light and dark conditions.

(a, b) GO terms enriched among genes upregulated (364 genes) or downregulated (655 genes) under –P versus +P in light.

(c, d) GO terms enriched among genes upregulated under dark versus light within +P (4668 genes) or –P (3751 genes) conditions.

(e, f) GO terms enriched among genes downregulated under dark versus light within +P (5712 genes) or –P (5127 genes) conditions.
