# Supplementary material for: Darkness Attenuates the Early Transcriptional Response to Phosphate Deficiency in Soybean Roots
Source: Int J Mol Sci. 2026 Sep 7;27(17):7963. doi: 10.3390/ijms27177963 (PMC13565979; doi:10.3390/ijms27177963)
Supplement: Supplementary file 1 [file ijms-27-07963-s001.zip › Supplementary_Table S2.pdf]

Supplementary Table S2. Genes with a significant light × phosphate interaction. Log_2 fold changes for −P versus +P are shown separately under light and dark conditions. The interaction log_2 fold change represents the P_i response in darkness minus the P_i response under light. Negative values indicate that the response to −P was reduced in darkness, whereas positive values indicate that the response was greater in darkness. Genes were considered significant at adjusted interaction $p < 0.05$; no additional fold-change threshold was applied.

**Gene** (annotation)	**log2FC (-P vs +P), light**	**log2FC (-P vs +P), dark**	**Interaction log2FC [dark (−P vs +P) – light (−P vs +P)]**	**Interaction adjusted p-value**
CHS4A (chalcone synthase 4A)	1.21	-2.90	-4.11	0.00257
LOC100306259 (uncharacterized)	-3.83	0.28	4.11	0.01160
LOC100815848 (uncharacterized)	1.68	-1.19	-2.87	0.01160
LOC100807179 (uncharacterized)	-0.70	1.21	1.91	0.01160
CHS1 (chalcone synthase 1)	1.43	-2.26	-3.70	0.03810
CHS7 (chalcone synthase 1)	0.43	-1.07	-1.49	0.04700
CHS12 (chalcone synthase 1)	0.34	-2.58	-2.92	0.04700
LOC102670430 (RING-H2 finger protein ATL56)	-1.57	0.29	1.86	0.04700
FVE (WD-40 repeat-containing protein)	-0.54	0.72	1.26	0.04700
CHS10 (chalcone synthase 1)	0.62	-2.19	-2.81	0.04970
SWEET5 (sugar efflux transporter SWEET5)	-0.94	4.11	5.05	0.04970
