# Supplementary material for: Darkness Attenuates the Early Transcriptional Response to Phosphate Deficiency in Soybean Roots
Source: Int J Mol Sci. 2026 Sep 7;27(17):7963. doi: 10.3390/ijms27177963 (PMC13565979; doi:10.3390/ijms27177963)
Supplement: Supplementary file 1 [file ijms-27-07963-s001.zip › Supplementary_Table S1.pdf]

Supplementary Table S1. Detailed Nanopore cDNA sequencing metrics for soybean roots under light and dark ±P conditions (four biological replicates per treatment). Values reflect per-replicate results before aggregation into Table 1.

condition	rep*	passed reads	mean lenth (bp)	N50 (bp)	mean Q score	GC (%)	QC-passed reads	mapped reads	mapping efficiency (%)	assigned reads	assigned (%)
**+P light**	1	5,732,265	1000	1,142	11	42.6	4,148,492	4,037,857	97.3	3,814,202	92
**−P light**	1	3,381,316	754	866	10	41.7	2,490,921	2,358,839	94.7	2,162,357	87
**+P dark**	1	4,933,634	663	727	10	42.3	3,389,997	3,265,584	96.3	2,861,462	84
**−P dark**	1	3,727,619	767	860	10	41.6	2,566,837	2,467,120	96.1	2,229,275	87
**+P light**	2	3,408,663	881	965	11	42.7	2,313,190	2,239,871	96.8	2,113,390	91
**−P light**	2	3,044,031	656	729	10	40.8	2,210,048	2,011,926	91.0	1,895,279	86
**+P dark**	2	9,915,490	950	1,007	11	41.9	7,014,401	6,836,685	97.5	6,496,583	93
**−P dark**	2	3,733,026	919	1,013	11	42.0	2,479,711	2,413,254	97.3	2,232,782	90
**+P light**	3	9,719,099	859	937	11	43.2	6,909,143	6,640,685	96.1	6,148,689	89
**−P light**	3	5,353,932	769	875	11	42.6	4,111,481	3,929,954	95.6	3,567,463	87
**+P dark**	3	4,537,328	695	775	10	42.7	3,221,989	3,069,511	95.3	2,567,450	80
**−P dark**	3	4,209,512	844	931	11	42.1	2,951,009	2,867,189	97.2	2,642,118	90
**+P light**	4	6,906,503	968	1,092	11	43.0	4,579,148	4,449,401	97.2	4,161,169	91
**−P light**	4	7,565,486	579	782	10	41.9	4,044,726	3,644,515	90.1	3,481,890	86
**+P dark**	4	7,255,796	794	1,008	10	43.9	4,622,765	4,318,145	93.4	3,755,442	81
**−P dark**	4	6,129,754	981	1,086	11	43.0	3,982,088	3,845,511	96.6	3,537,114	89

*rep = biological replicate.
